# Inactivation of microorganisms in the complex regions of transvaginal ultrasound probes by a UVC-LED light based disinfection system

**DOI:** 10.1371/journal.pone.0346554

**Published:** 2026-11-05

**Authors:** Muhammad Yasir, Mark D. P. Willcox

**Affiliations:** School of Optometry and Vision Science, University of New South Wales, Sydney, Australia; Universidade Estadual de Ponta Grossa, BRAZIL

## Abstract

Endocavity ultrasound transducers, particularly transvaginal ultrasound (TVUS) probes, contain intricate structures such as notches, grooves, lens surfaces, and handle edges that are highly susceptible to microbial contamination yet difficult to disinfect using conventional high-level disinfection (HLD) methods. This study evaluated the efficacy of a novel ultraviolet C light emitting diode (UVC-LED) HLD system in eliminating microbial contamination from these complex probe surfaces. Two TVUS probes were sampled from predefined high-risk regions before and after disinfection following clinical use. Probe A was sampled at the top and bottom notches and both sides of the handle, while Probe B was assessed at the lens, edges, and bent groove regions. Microbial contamination was quantified using swab sampling, culture on agar plates, and identification via Matrix-Assisted Laser Desorption/Ionization Time-of-Flight (MALDI-TOF). Environmental sampling of examination and disinfection rooms was also performed. To assess this system robustness, probe sites were repeatedly inoculated with *Bacillus subtilis* spores and evaluated following UVC treatment. Before UVC treatment, contamination rates ranged from 25% to 57% across sampled regions, with microbial loads reaching up to 3.9 log_10_ colony forming units (CFU). Identified organisms included *Staphylococcus epidermidis,* *Pseudomonas koreensis*, *Bacillus cereus,* and *Propionibacterium* spp. Probe sheaths were also predominantly contaminated with *Staphylococcus epidermidis*., with counts reaching up to 4.3 log_10_ CFU, Environmental sampling revealed diverse microbiota, with higher contamination levels in examination rooms compared to disinfection areas. Following 90 seconds of UV-C exposure, no microbial growth was detected on any sampled site, indicating 100% decontamination. Additionally, UVC treatment achieved > 6.5 log_10_ reduction of *B. subtilis* spores across all tested regions. These findings demonstrate that UVC-LED technology provides rapid, effective, and consistent high-level disinfection of complex TVUS probe surfaces, supporting its potential as a rapid and reliable disinfection modality in clinical setting.

## Introduction

Ultrasound transducers (also known as probes) are used for a variety of diagnostic and procedural applications, including transvaginal (TV), transrectal (TR), transoesophageal echocardiography (TOE), and imaging of open wounds. During these procedures, the transducer may come into contact with mucous membranes of the vagina, rectum, oropharynx and oesophagus, as well as non-intact skin such as ulcers or open wounds. Importantly, when they are used in sterile body areas, such as the intravascular space, there might be high chances of risk of infection. Certain structural features of ultrasound probes, including notches, grooves, seams, and textured surfaces, may increase the risk of contamination. The probe handles may also remain contaminated, potentially posing a risk to patients [1]. These areas can retain biological material or microorganisms, if not properly disinfected.

Inadequate reprocessing of transvaginal ultrasound (TVS) probes can lead to cross-contamination and nosocomial infections, posing a serious risk to patients’ health, in particular to pregnant patients [2,3]. Commonly, the vaginal microbiota such as *Gardnerella vaginalis, Ureaplasma urealyticum, Candida albicans, Prevotella* spp., and *Lactobacillus* spp have been found associated with probes infection [4,5]. Pathogens not usually associated with the normal microbiota of the healthy vagina including human papillomavirus [6], Epstein Barr virus 9 [7], human immuno deficiency virus (HIV), cytomegalovirus, Gram-negative bacteria such as *Escherichia coli*, *Pseudomonas aeruginosa* and *Klebsiella* spp. Gram-positive bacteria such as methicillin-resistant *S. aureus*, *Clostridium difficile,* vancomycin-resistant *Enterococcus* spp, *Neisseria gonorrhoeae, Treponema palladium*, and *Mycobacterium avium* can also contaminate the probes and may cause disease [8].

Probe contamination may also occur due to perforation, tearing, or leakage of the probe cover during use, allowing body fluids or microorganisms to contact the probe surface. This can account for up to 9.0% of infections [9–11]. Reported leakage rates range from 0.4% to 13% for condoms and 0% to 5% for commercial covers [12]. Additionally, there is an estimated 9% risk of condom perforation in patients undergoing transrectal biopsy under ultrasound guidance [11]. Similarly, perforation rates of up to 81% have been reported for commercial probe covers, resulting in contamination of transducers with blood and other body fluids [9,11].

Various methods, including chemical soaking, chemical aerosolisation, and surface wiping, have been employed for ultrasound probe disinfection in IVF (in vitro fertilization) clinic. Chemical soaking requires the probe to be immersed in a disinfectant solution for approximately 8–45 minutes, whereas chemical aerosolisation involves nebulisation of hydrogen peroxide within a device chamber and may require approximately 7–12 min, depending on the system and operating conditions. Quaternary ammonium compounds and chlorhexidine in solution [6], or soaked with wipes [13] found ineffective against HPV. Similarly, glutaraldehyde exhibits broad-spectrum antimicrobial activity, but its use is limited due to potential damage to ultrasound probes [14]. These chemical-based approaches may present occupational and environmental concerns due to potential exposure to chemical disinfectants.

Most ultrasound transducers cannot undergo established sterilisation processes; therefore, it is imperative that correct high-level disinfection (HLD) is undertaken in IVF clinic. In the clinical workflow. turnaround time between patients is an important consideration, particularly in high-throughput environments. Routine IVF ultrasound examinations are typically brief (5–10 min), requiring rapid reprocessing of probes between patients. The Lumicare UVC-LED disinfectant system is designed to provide HLD within a timeframe compatible with clinical workflows, enabling probe availability for subsequent patients without significant disruption to clinic throughput. In a previous study [7], the Lumicare HLD disinfectant system disinfected transvaginal ultrasound probes (TVUS) within 90 sec and effectively eliminated microorganisms associated with them. However, to reinforce its effectiveness, the current study focussed on the most challenging probe areas (notches, seams, grooves, and textured surfaces), commonly referred to as potential cold spots of the probes (cold spots being defined as areas where light, including ultraviolet light may not easily reach). Therefore, the current study assessed the effectiveness of a UVC system in reducing microbial load in the complex areas of two different types of ultrasound probes.

## Methods

### *Ex vivo* testing

This study was approved by the Human Research Ethics Committee of the University of New South Wales (HREC number: HC220136). Following Inclusion/Exclusion Criteria were used to recruit the participants.

- 18 years of age or older
- Attending Connect IVF for a transvaginal ultrasound test
- No known or suspected vaginal infections

Participants who meet the following criteria will be excluded from the study:

- Under the age of 18 years
- People highly dependent on medical care who may be unable to give consent
- People with cognitive impairment, an intellectual disability, or a mental illness

The ultrasound probes (Mindray, China, Fig 1) were collected (from total thirty subjects) after use in female (aged >18 years) an in vitro fertilization clinic (Connect IVF, Sydney, Australia) from February 26 to April 02, 2025. All the subjects completed the study. Prior to use, these probes had been sheathed with a non-sterile latex free probe cover (Sonologic), on which visible debris was present (standard ultrasound gel was applied to the transducer tip before covering it with the sheath to ensure proper coupling for ultrasound transmission). After use, the sheath was removed, and the probes were sampled immediately with sterile cotton swabs (Bacto Laboratories, Mount Pritchard, NSW, Australia) by passing twice over a 2–5 cm^2^ area (initially marked to facilitate identification) from the probes including from the top notched, bottom notched, left side of the handle, right side of the handle of one probe, grooves and lens areas of other probe (randomly selected) to examine the numbers and types of microbes present on the endoscopes prior to disinfection. All the sampling was performed by a single IVF clinic staff trained by the UNSW researchers in accordance with the standard operating procedures. The probe’s area on was initially marked to facilitate identification and was subsequently applied consistently to all probes throughout the study. The reproducibility of sampling could not be determined due to the nature of the sampling. The standard cleaning of ultrasound probes was involved a basic rinse with water and wipe over with a paper towel prior to HLD. However, no wipe-down or manual cleaning step was performed between removal of the sheath and swab sampling, this was a deliberate study design choice to capture worst-case contamination. The probes were then loaded into the Lumicare ONE High Level Disinfection UVC system (Lumicare 606A743U7Y, Sydney, Australia; Fig 2) and the system run for a standard time of 90 sec. During a standard HLD cycle, UVC LEDs generate UVC irradiation (265 nm – 275 nm) across the entire surface of the ultrasound probe positioned within the enclosed disinfection chamber at an approximate distance of 3 cm. After disinfection, the ultrasound probe was removed, and the opposite side of each probes’ sites was swabbed as described above. The swabs were then put into AMIES transport media (Bacto Laboratories, Australia) and transported to the microbiology laboratory of the School of Optometry and Vision Science University of New South Wales (UNSW) in cold chain and processed within 4 h of collection.

**Fig 1:**
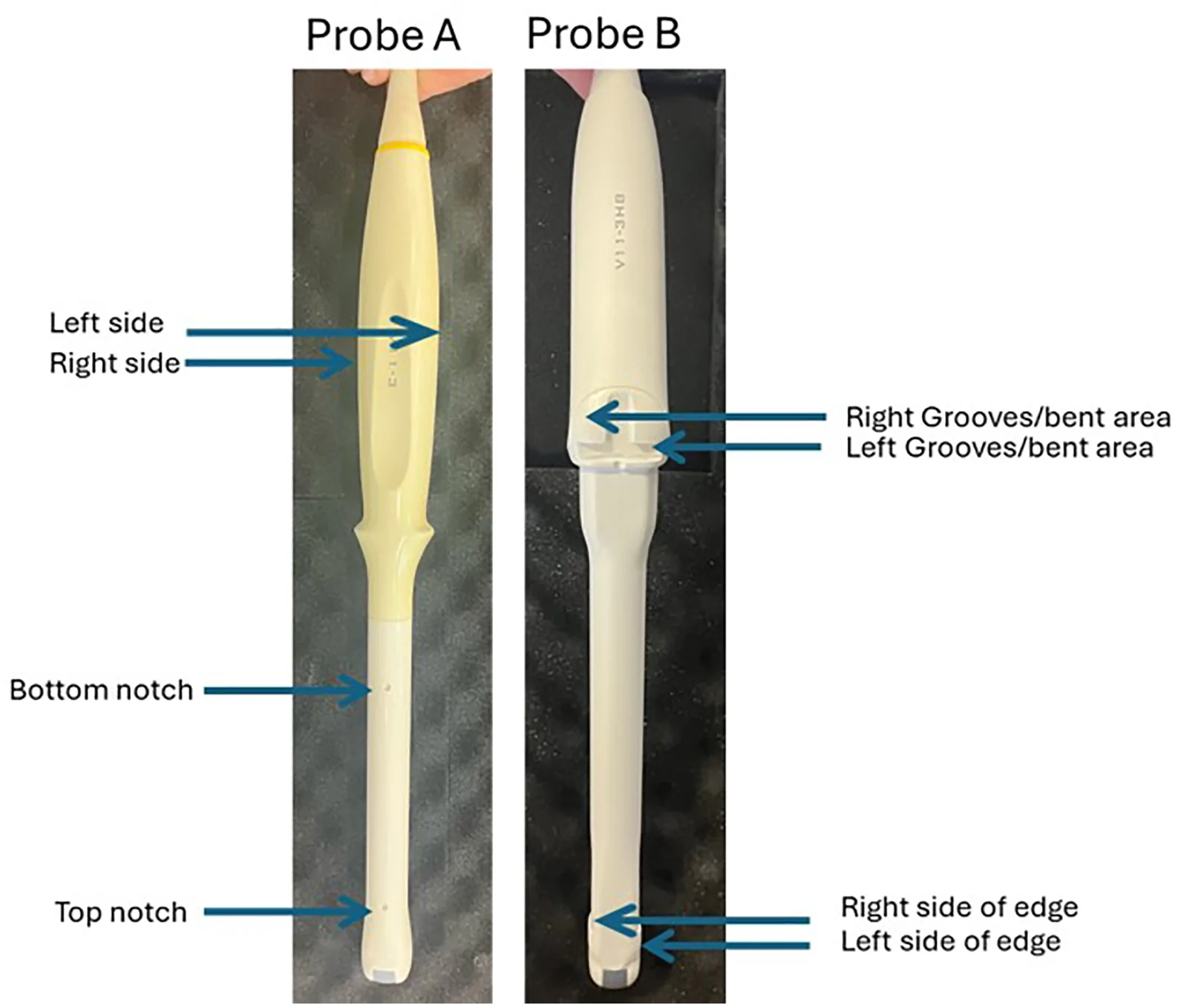
Ultrasound probes used in the current study. The blue arrow pointed the areas from where samples were swabbed before and after UVC treatment for isolation of microorganisms.

**Fig 2:**
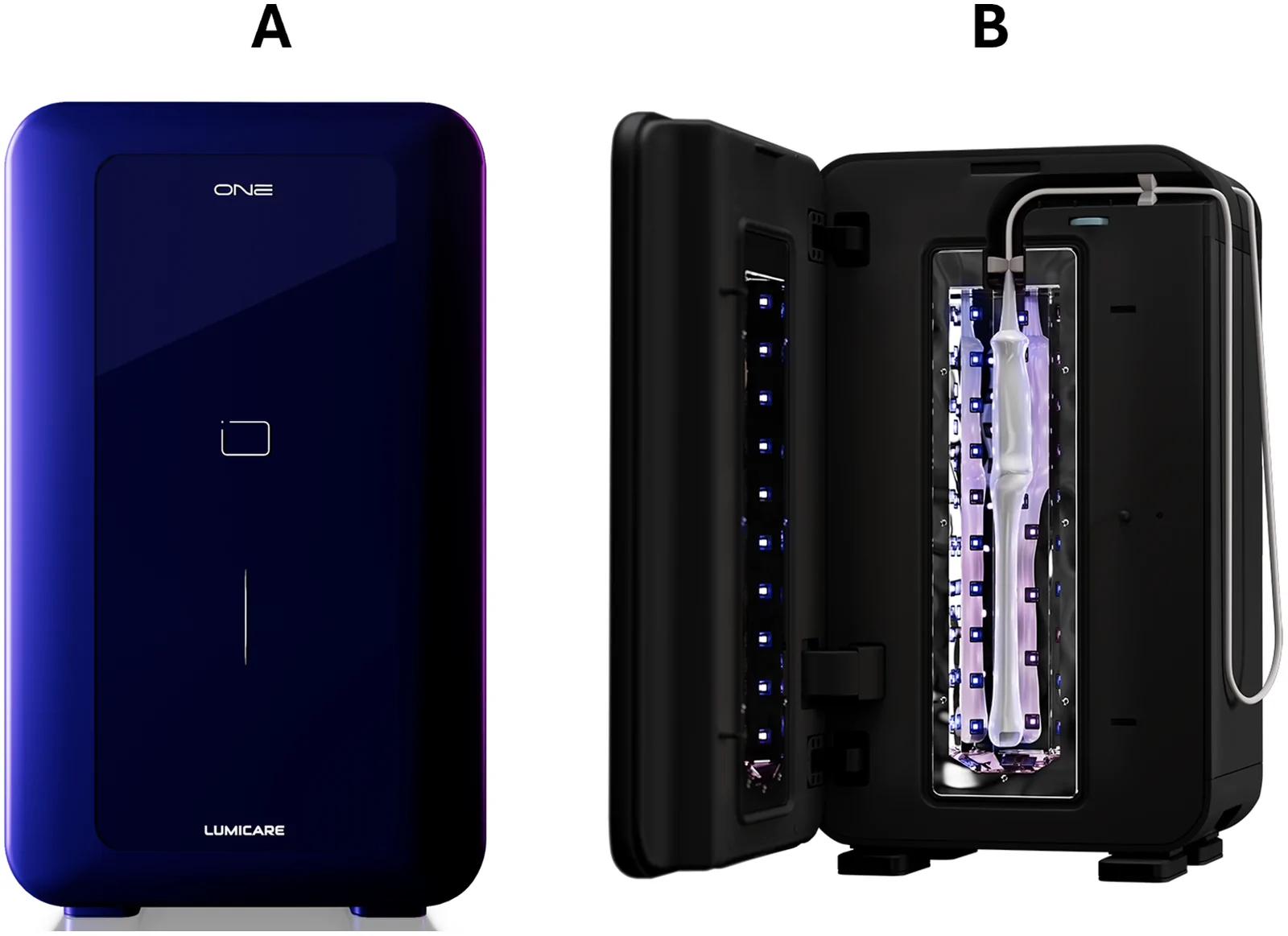
Lumicare UVC-LED based system: frontal view (A) and chamber view showing the probes suspended inside the chamber (B).

To assess whether the sheaths (protective covering of ultrasound probes) had any bacterial contaminations, five sheaths were randomly selected and analysed for the presence of contaminants. The sheaths were swabbed from the internal surfaces over a 5–10 cm^2^ area which had directly contacted the probes.

All the swabs were processed under aseptic conditions. Microorganisms were recovered in Phosphate Buffer Saline (PBS); NaCl 8 g/L, KCl 0.2 g/L, Na_2_HPO_4_ 1.4 g/L, KH_2_PO_4_ 0.24 g/L; pH 7.4)) by vortexing the swabs in vials in the presence of glass beads (0.5 mm diameter, Sigma Aldrich St Louis, MO, USA) for 3–5 min. 200 µL of the resulting slurry was spotted onto chocolate blood agar (CBA); Beckton Dickinson, Maryland, USA)) and incubated in three different atmospheric conditions (aerobic, microaerophilic and anaerobic) for 24–72 h at 37^o^C. 200 µL was also plated onto Sabouraud’s dextrose agar (SDA); Beckton Dickinson, Maryland, USA)) and incubated for 5–7 days at 25^o^C to grow any fungi present on the endoscopes. Following incubation, numbers of similar colonies of each bacterium and fungus were counted and purified. Pure cultures of microbes were stored in 25% glycerol at −80 °C.

### Environmental sampling

To assess whether there were any microorganisms (bacteria, fungi, yeasts) present in the environment during the process of sampling and disinfection, environmental monitoring was assessed using agar plates. One plate of each CBA and SDA with opened lids were placed in the clinical and disinfectant rooms for the entire duration of sample collection and disinfected in the Lumicare device.

### Microbial identification

Microbes were recultured on CBA and incubated overnight. A loopful of growth was suspended in 300 μL of HPLC-grade water, mixed, and treated with 900 μL absolute ethanol. After centrifugation (13,000 rpm, 2 min) and drying, pellets were dissolved in 25 μL of 70% formic acid and 25 μL acetonitrile, then centrifuged again. One μL of the supernatant was spotted on a MALDI plate, overlaid with α-cyano-4-hydroxycinnamic acid (HCCA) matrix, air-dried, and analysed by MALDI-TOF for microbial identification [15].

### *In vitro* testing

#### Sterilising the probes.

Two ultrasound probes with notches at the top and middle, one bent at the centre were used in this study. To confirm that the ultrasound probes were sterile at the start and after every disinfection cycle, they were disinfected with 4% sodium hypochlorite (bleach) for 4–5 min before each *Bacillus* spore inoculation, and then dried for 20–30 min. After decontamination with bleach, the probes were rinsed with sterile Milli-Q water to remove any residual bleach and prevent interference in subsequent use.

### Spore testing

The spores of *Bacillus subtilis* ATCC 19659 were produced following a published method [7]. Then spores in suspension (1 × 10^9^ spores/mL; 20 μL) were dried for 30–45 min on probe notches, bends, and transducer lenses. Probes were then either left at room temperature (22–25°C) or exposed to UVC for 90 sec using the Lumicare ONE HLD system. To get the consistent effectiveness against all the probes, same machine was used for both *ex vivo* and *in vitro* testing. Following treatment, spores were recovered by swabbing the inoculated areas. Swabs were vortexed in PBS containing glass beads for 3–5 min, followed by serial dilution and plating onto TSA. Plates were incubated at 37 °C for 24 h. Similarly, spores were recovered from control areas of the same surface that did not receive UVC treatment. Uninoculated areas were processed using the same procedure to confirm sterility. No neutralising agent was required, as UVC exposure does not result in residual antimicrobial activity.

### Statistical analysis

The numbers and type of sites of the transvaginal ultrasound probes after use ex vivo and recovery of *Bacillus* spore in vitro before and following a disinfection cycle in the Lumicare ONE High Level Disinfection device was compared using nonparametric (Mann Whitney test) with Graph Pad Prism version 11.0.2.(92). Due to the small sample size, the data were not assessed for normal distribution and were therefore analysed using non-parametric statistical tests. A P value less than 0.05 indicated statistical significance.

## Results

### *Ex vivo* testing

To demonstrate the level of contamination on transvaginal ultrasound probe, various areas of the probes were swabbed and examined for the presence of bacteria. Different areas of probes A and B were heavily contaminated with microbes before UVC treatment. A diverse range of bacterial species was detected on each probe. For probe A, these included *Staphylococcus, Pseudomonas, Propionibacterium, Bacillus*, *Legionella*, and *Tsukamurella* spp. Approximately, all sites of the probe were contaminated with multiple bacterial species with the range of contamination from 1.6 log_10_ CFU to 3.7 log_10_ CFU (Table 1).

**Table 1:** Numbers of identified bacteria on different sites of probes A and B before treatment with UVC.

Patient ID	Top Notch untreated (log_10_ CFU)	Bottom Notch untreated (log_10_ CFU)	Right side of handle untreated (log_10_ CFU)	Left side of handle untreated (log_10_ CFU)
**Probe A**				
**Sample 1**	• *S. epidermidis* (1.6) • *P. fulva* (1.6)	Nil	Nil	Nil
**Sample 2**	• *Propionibacterium* spp. (3.5)	Nil	Nil	Nil
**Sample 3**	• *P. fulva* (3.5)	Nil	Nil	Nil
**Sample 5**	Nil	Nil	*S. warneri* (2.6) *S. lugdunensis* (2.3)	Nil
**Sample 6**	Nil	Nil	*S. warneri* (2.9) *L. pneumophila* (1.8)	Nil
**Sample 9**	*S. epidermidis* (2.2)	*S. epidermidis* (2.7)	Nil	*Pseudomonas balearica* (3.1) *B. amyloliquefaciens* (3.7)
**Sample 10**	Nil	*S. epidermidis* (2.7)	Nil	
**Sample 15**	Nil	Nil	*Tsukamurella hongkongensis* (3.1)	Nil
**Probe B**				
**Sample 17**	*Legionella pneumophila* (2.8)	Nil	Nil	Nil
**Sample 18**	*B. cereus* (3.6)	Nil	*P. koreensis* (3.8)	Nil
**Sample 19**	Nil	Nil	*S. epidermidis* (1.8)	Nil
**Sample 20**	*B. cereus* (2.1)	Nil	*S. epidermidis* (3.3)	Nil
**Sample 22**	Nil	Nil	*Bacillus infantis* (3.5)	Nil
**Sample 23**	Nil	Nil	Nil	*S. capitis* (1.9)
**Sample 24**	Nil	Nil	Nil	*B. cereus* (3.0)
**Sample 25**	Nil	*S. epidermidis* (2.3)	Nil	Nil
**Sample 28**	Nil	*S. epidermidis* (3.0)	Nil	Nil
**Sample 30**	Nil	*S. epidermidis* (3.9)	Nil	Nil

Similarly for probe B, a variety of bacterial species were detected, including *Legionella pneumophila*, *Staphylococcus*, *Pseudomonas,* and *Bacillus* spp. Almost all sites on the probe were contaminated with different bacterial species with the range of contamination from 1.8 log_10_ CFU to 3.9 log_10_ CFU) (Table 1).

Rate of contamination of specific site was calculated based on the formula. (Number of site positive for microbial growth/ total number of sites screened for bacterial growth × 100). While 100% decontamination for specific site was defined based on the formula (Number of site negative for microbial growth/ total number of sites screened for bacterial growth ×100). The rate of contamination of top notches of the probe was 57% (4/7), bottom notches 25% (2/8), right side of handle 43% (3/7), left side of handle 25% (2/8) before UVC treatment (Table 2). However, after UVC treatment, rate of contamination was 0% of all tested sites of probe (Table 2). Similarly, Probe B was also heavily contaminated with microbes before UVC treatment. All the sites shown (Table 2), were assessed for microbial presence and found contaminated on non-UVC treated sites. Rate of contamination of right side of edge 43% (3/7), left side of edge 38% (3/8), right side of groove 57% (4/7), left side of groove 25% (2/8). However, after UVC treatment, there was complete killing of microorganisms on each treated site, and no microbial growth was detected on any treated site. The rate of contamination after UVC treatment on all the sites of probe was 0% (Table 2).

**Table 2:** Sites of probes A and B showing bacterial contamination before and after UVC treatment.

Location	Before UVC	% Contaminated Before UVC	After UVC	% Contaminated After UVC	P value
**Probe A**					
Top Notch	4/7	57	0/7	0	< 0.05
Bottom Notch	2/8	25	0/8	0	
Right Side of Handle	3/7	43	0/7	0	
Left Side of Handle	2/8	25	0/8	0	
**Probe B**					
Right Side of Edge	3/7	43	0/7	0	< 0.05
Left Side of Edge	3/8	38	0/8	0	
Right Side of Groove	4/7	57	0/7	0	
Left Side of Groove	2/8	25	0/7	0	

Probe covers are often utilized to prevent contamination of probes, but they could also serve as a source of infections. Some of the probe sheaths used for covering the probes were found to be contaminated. No microbial growth was detected on sheath 1. All other sheaths showed contamination with different bacterial species including *S. epidermidis, S. lugdunensis, B. oceanisediminis***,** *Nocardioides simplex, Corynebacterium aurimucosum* with the range of contamination from 3.3 log_10_ CFU to 4.3 log_10_ CFU) (Table 3).

**Table 3:** Numbers of bacteria identified on the sheaths (protective covering of probes).

Covering	Bacterial type	Log_10_ CFU
Sheath 1 (from sample 10)	*Nil*	0
Sheath 2 (from sample 17)	*S. epidermidis*	3.6
Sheath 3 (from sample 21)	*S. lugdunensis* *B. oceanisediminis*	3.5 3.4
Sheath 4 (from sample 24)	*S. epidermidis*	4.3
Sheath 5 (from sample 29)	*N. simplex* *C. aurimucosum* *S. epidermidis*	4.2 3.9 3.3

### *In vitro* testing

In order to determine the efficacy of UVC treatment, sodium hypochlorite (4%) sterilized probes were inoculated with control bacillus spores and the reduction in recovered spores were measured before and after UVC treatment. No spores germinated from the probes after disinfection with 4% bleach, indicating that this method was effective for decontaminating probes prior to use in the study as before inoculation of each repetition. In addition, no visible bacterial growth was observed in the sterile recovery broth after incubation for up to 24 h, confirming that the broth remained sterile. On other hand, visible growth was observed in the tube containing 1.0 mL of a 1 × 10⁷ spore dilution, demonstrating that the spores were viable. Spore suspensions adjusted to OD_620_ values of 1–3 yielded 1.5 × 10^9^ CFU/mL in hard water containing 5% horse serum.

The efficacy of UVC treatment in reducing microbial contamination on the complex probe sites such as handle, notch, and lens, was evaluated by comparing viable counts recovered from control and UVC treated samples following five consecutive inoculations with Bacillus spores. Before UVC treatment, mean recoveries of 6.94 ± 0.04 log_10_, 7.12 ± 0.15 log_10_, and 6.82 ± 0.12 log_10_ CFU were observed on the handle, notch, and lens areas of probe A, respectively. Following UVC exposure, recoveries were reduced to 0.14 ± 0.31 log_10_, 0.20 ± 0.45 log_10_, and 0.00 ± 0.00 log_10_ CFU on the corresponding sites. Overall, UVC treatment resulted in reductions of 6.80 log_10_ on the handle, 6.92 log_10_ on the notch, and 6.82 log_10_ on the lens of probe A (Fig 3A). Similarly, for probe B, untreated control samples showed mean recoveries of 6.99 ± 0.08 log_10_, 7.11 ± 0.40 log_10_, and 6.85 ± 0.08 log_10_ CFU on the central bent side, left edge, and lens area, respectively. After UVC treatment, viable counts decreased to 0.20 ± 0.45 log_10_, 0.14 ± 0.31 log_10_, and 0.00 ± 0.00 log_10_ CFU on the same sites. Consequently, UVC exposure resulted in log_10_ reductions of 6.79 on the central bent side, 6.97 on the left edge, and 6.85 on the lens area of probe B (Fig 3B).

**Fig 3:**
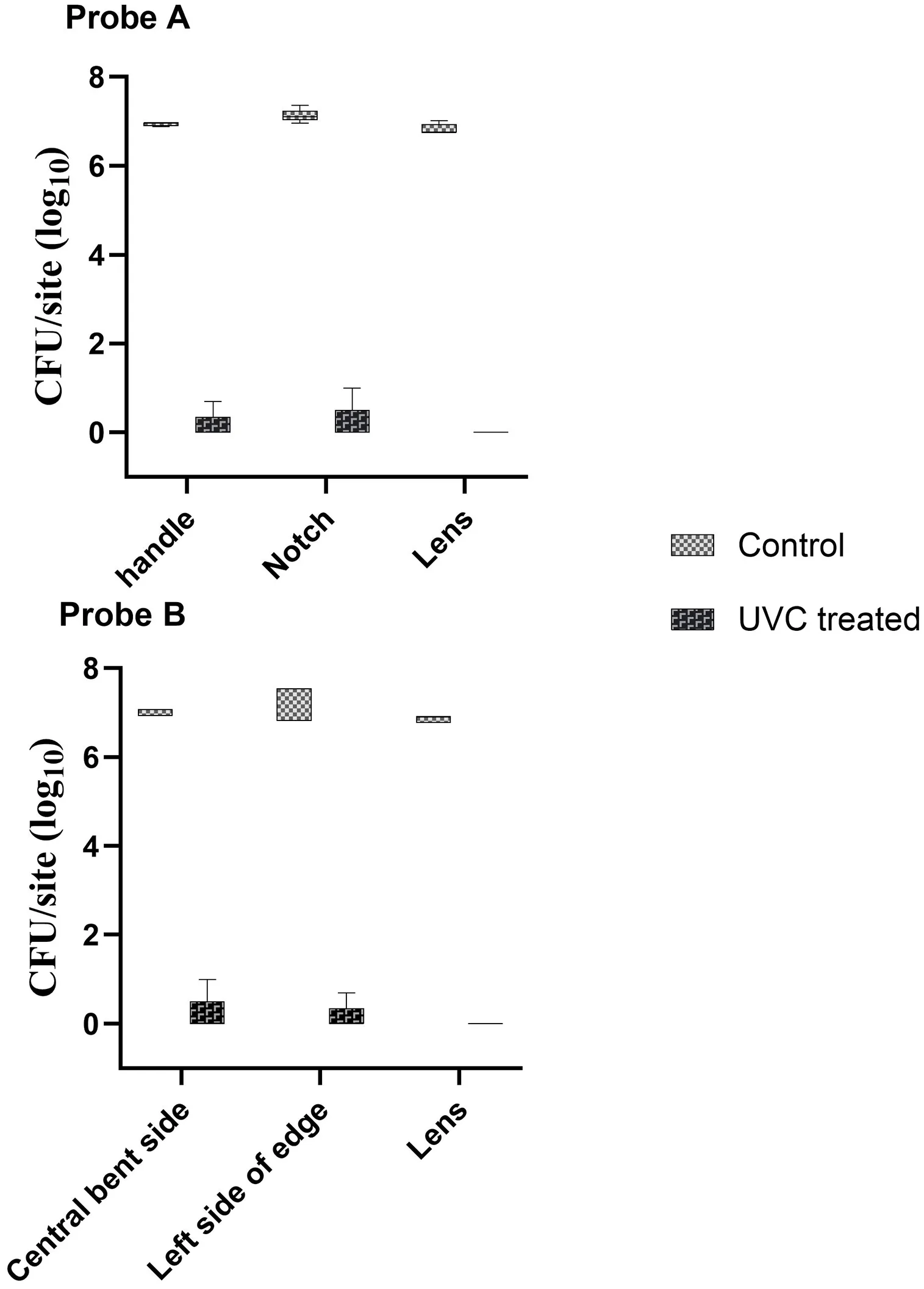
Effect of UVC treatment on recovery of *B. subtilis* spores from different sites of probes. Spore viable counts (log_10_ CFU/site) recovered from control (non UVC treated) and samples (UVC treated) are shown for the handle, bottom notch and lens (**Probe A)**, and central bent side, left side of edge and lens of **(Probe B**). Recovery of spores from control sites were > 6.5 log_10_ CFU/site, however after UVC treatment recovery of spores from samples sites decreased to < 0.5 log_10_ CFU/site (p < 0.05). This shows that UVC-LED system reduced > 6 log_10_ bacillus spores on complex sites of both probes.

Overall, UVC treatment resulted in complete inactivation of recoverable microorganisms on both probes and handle, bent areas, notch, edges and lenses, achieving reductions of approximately > 6 log_10_ CFU/site when compared with untreated controls.

### Environmental sampling

The environments of both the clinical room (where samples were collected) and the disinfectant room (where samples were processed using the UVC device) were found to be contaminated throughout the study period. Overall, the clinical room tended to show higher levels of contamination than the disinfectant room on each day, with the types of bacteria found varying between days and rooms (Supplementary Table 1).

## Discussion

The present study demonstrates that probes can be extensively contaminated prior to disinfection, with multiple complex sites harbouring diverse and clinically relevant bacterial species. Before UVC exposure, contamination rates ranged from 25% to 57% across different probe sites including notches, handles, edges, and grooves. However, UVC-LED system effectively reduced the microbial load by killing 100% on all these complex sites after treatment with standard cycle for 90 sec. The efficacy of UVC-LED was not affected by the microbial contaminants found in the environment where these probes have been disinfected.

Complex probe geometries such as depressed or irregular areas (notches and grooves and bent areas) provide favourable niches for microbial retention and are difficult to clean [16]. In this study, two probes featuring such notches and grooves were examined to assess microbial contamination following clinical use and subsequent disinfection using a UVC-LED system. These recessed areas provide sheltered sites that hinder routine cleaning and disinfection because of surface irregularities and limited accessibility. A diverse microbial community, including *Staphylococcus*, *Pseudomonas*, *Propionibacterium*, *Bacillus*, *Tsukamurella* spp., and *L. pneumophila*, was recovered from these hidden regions on untreated probes. Similar organisms have previously been reported on various probe surfaces [1,17]. The probe’s handle is another site which can become contaminated with different microorganisms. *Pseudomonas* spp*, Staphylococcus* spp*,* and *L. pneumophila* can be transferred from environmental surfaces or the skin of handling staff to the probes [18]. Additionally, two *Bacillus* species were found on the probes’ handles or notches (lens) consistent with earlier study Ejtehadi et al [17],. *Bacillus* are spore forming bacteria and represent stable environmental reservoirs contributing to probe contamination.

According to the Australasian Society for Ultrasound in Medicine (ASUM), Australian Standard (AS5369), and American Institute of Ultrasound in Medicine (AIUM) guidelines, transvaginal ultrasound probes are classified as semi-critical devices and require HLD between patients [19–21]. Various disinfectants have been used for probe disinfection, including quaternary ammonium compounds, chlorhexidine, and quaternary ammonium-based wipes. Although these agents are effective against a range of bacteria and fungi within 5–20 min, their effectiveness against certain viruses, including Human papillomavirus (HPV), may be limited [6,13]. Sherman et al. [22] reported that 70% alcohol was highly effective against bacterial contamination but demonstrated limited activity against viral contamination. Similarly, glutaraldehyde, ortho-phthalaldehyde, and 4% chlorhexidine gluconate were found to be ineffective against HPV16 [23,24]. However, UV-C devices can disinfect vaginal ultrasound probes faster and can inactivate all the microorganism including viruses [25,26]. The current study demonstrated that following treatment (90 sec) with the Lumicare UVC-LED system, no microbial growth was detected on any of the treated areas of the probes. This suggests that UVC-LEDs delivered UVC light effectively across the entire probe surface using UVMESH technology, resulting in the elimination of microorganisms from complex and uneven probe surfaces [27]. A previous study reported that 3D scanning of endocavity bi-plane and curved array probes demonstrated an uneven distribution of irradiance across probe surfaces when exposed to a UVC system [28], which may compromise the performance of the disinfectant system. However, the current UVC-LED device incorporating UVMESH technology achieved complete microbial inactivation, including on complex region of both probe types. This difference may be attributed to variations in study design, as the previous study primarily modelled or assumed irradiance distribution across differently designed probe surfaces rather than evaluating the real-world antimicrobial efficacy of the disinfection system. In contrast, the present study directly assessed microbial inactivation on probe surfaces under practical conditions. Additionally, probe design and geometry can also contribute to variations in irradiance distribution across specific probe sites [28]. The UVMSH technology enhances spatial distribution and more uniform irradiance on entire area of target surfaces. Moreover, this approach specifically can minimise the risk of small areas remaining untreated and maximises decontamination efficiency, even across complex surface architectures and geometries. Additionally, the system is engineered to provide controlled and reproducible UVC exposure conditions, potentially enhancing consistency in microbial inactivation outcomes. *Staphylococcus* species particularly *S. epidermidis*, *S. haemolyticus*, and *S. warneri* were frequently recovered from complex probe sites. Notably, *S. epidermidis* reached levels of 3.9 log_10_ CFU on the left side of the top notch of untreated probes. This location is known to be one of the most consistently contaminated areas of ultrasound probes. Previous studies also determined the presence of similar numbers of *Staphylococcus* spp. on probe surfaces which were reduced by UVC-LED system within 90 sec [7]. In another study, a UVC device achieved a 2.17 log_10_ reduction of *S. aureus*, *E. faecalis,* and *E. coli* within 5–7 min when disinfecting probes [29]. The longer disinfection time (5–7 min) reported in the previous study, compared with the 90 sec cycle in the recent study, may be attributed to probe disinfection being performed in the presence of ultrasound gel. In contrast, the gel was completely removed prior to disinfection in the present study. The presence of gel may have impeded UVC penetration, thereby reducing disinfection efficiency and necessitating a longer exposure time. Given that several microorganism including *Staphylococcus* spp. are common skin commensals [30], they can easily transfer from human skin to probes during handling. Therefore, minimising unnecessary contact after HLD is essential to prevent reintroduction of microorganisms into these difficult to access probe areas which can be done by storing them properly in protective covers.

Although probe covers designed to act as protective barriers, can themselves become contaminated during ultrasound procedures through contact with mucous membranes, infectious sites, or open wounds [26]. In the present study, four of five sheath samples were contaminated with *B. oceanisediminis*, *S. epidermidis*, *S. lugdunensis*, *N. simplex*, and *C. aurimucosum*. Previous work has shown that probe covers can transfer microorganisms to probes, serving as an additional contamination source [26]. Previous study demonstrated that probe covers were contaminated with *B. subtilis* with level up to 2.8 log_10_ [7]. Detection of high level (4.3 log_10_ CFU) of *S. epidermidis* on probes sheaths in recent study suggests compromised barrier integrity, improper handling, or environmental contamination during application or removal. Similarly, detection of multiple species on individual covers further indicates that the use of sheaths alone does not eliminate contamination risk and underscores the continued need for effective disinfection protocols even when protective barriers are used. Consistent with earlier recommendations, a new condom or probe cover should be applied for every patient and for each use of the instrument [31].

The performance of UVC-LED system was also evaluated in vitro using Bacillus spores. In vitro validation confirmed the robustness of the experimental design. The absence of spore germination following 4% bleach treatment verified that decontamination was effective prior to inoculation experiments as demonstrated earlier [7]. However, this method is not recommended by probe manufacturers due to the potential for damage to the metal and plastic components of the probes [21]. Recovery of viable *B. subtilis* spores and sterile control media further supported the accuracy of microbial reduction measurements. UVC-LED system effectively eliminated microorganisms across all probe sites tested. Under high‑level *Bacillus* spore challenge conditions, UVC exposure achieved > 6 log_10_ CFU reductions on all tested sites, suggesting effective penetration and coverage despite geometric complexity of the probes. Such reductions meet accepted standards for high‑level disinfection, even against highly resistant spore‑formers. The small spore counts (0.23–0.33 log_10_ CFU) may be attributable to the detection of organisms near the assay’s detection limit. In this study *Bacillus* spp. was selected because related several *Bacillus* spp were isolated from used probes. Additionally, *Bacillus* spores are internationally recommended test organisms (EN12353, EN14561, AOAC 966.04), and they exhibit exceptional resistance to disinfection [15,16].

The combined clinical and laboratory findings highlight two major implications. First, routine probe use in clinical environments can result in substantial and diverse microbial contamination, even when protective sheaths are utilized. Second, UVC disinfection provides rapid and highly effective microbial reduction, including against resistant spore-forming bacteria, making it a strong candidate for high-level disinfection of complex reusable probes with varying structural complexities without causing any damage to the probes. As part of the regulatory evaluation of the Lumicare UVC-LED system, material compatibility testing confirmed that repeated exposure to UVC does not adversely affect probe integrity or performance. Lumicare device has completed material compatibility assessments through its regulatory pathways (CE Mark and ARTG registration), and compatibility has been confirmed with major ultrasound probe manufacturers, including the manufacturers of the probes evaluated in this study. These assessments demonstrated that repeated UVC-based high-level disinfection does not result in detrimental effects on probe materials.

Environmental monitoring over four days showed higher microbial loads in the clinical room than in the disinfection room, with several microorganisms also detected on probe surfaces. The recovery of *Staphylococcus,* *Bacillus*, *Pseudomonas*, and *L. pneumophila* suggests potential environmental contribution to probe contamination through handling, surface contact, or moisture-associated routes [30,32]. While environmental contamination was documented but its transmission dynamics was not determined, future research should investigate the transmission dynamics with an SAS 180 Impaction Air Sampler.

This study has several strengths, including site-specific contamination mapping, identification of a broad spectrum of microorganisms, environmental monitoring, and rigorous spore-based challenge testing. However, certain limitations should be acknowledged for the recent study. Viral pathogens were not evaluated in the recent study. A previous study reported the detection of HPV on vaginal ultrasound probes and demonstrated that the virus was completely inactivated by a UVC disinfection system within a 90 sec disinfection cycle [26]. Future research should explore the effectiveness of Lumicare UVC-LED system against viral pathogens especially against HPV and spores of the *C. difficile* (a faecal contaminates). Another key limitation of this study was the inclusion of only two probes from a single probe category. Ultrasound probes are broadly classified into endocavity probes (e.g., transvaginal and transrectal) and external probes (e.g., linear, curved, and phased array) [33]. The present study evaluated the effectiveness of the Lumicare UVC-LED system only against endocavity probes, using representative commonly used transvaginal/transrectal ultrasound probes manufactured by Mindray (China). However, each probe type contains unique and complex surface geometries that require independent evaluation for effective disinfection. Consequently, this study provides limited insight into the disinfection performance across the broader range of ultrasound probe types. Therefore, future studies should evaluate the effectiveness of the Lumicare UVC system on multiple probe types with varying structural complexities.

In conclusion, the findings demonstrate that the Lumicare UVC-LED system can effectively disinfect both straight and curved transvaginal probes by inactivating microorganisms residing in complex probe sites such as notches, handle, grooves, and lens areas etc. Furthermore, UVC treatment achieved consistent and substantial (> 6 log_10_) reduction of *B. subtilis* spores across all tested complex sites, highlighting its potential to inactivate highly resistant microorganisms.

## Supporting information

S1 Table Numbers of bacteria identified in the environment where probes were sampled and processed. (DOCX)
