# Supplementary material for: Inactivation of microorganisms in the complex regions of transvaginal ultrasound probes by a UVC-LED light based disinfection system
Source: PLoS One. 2026 Nov 5;21(11):e0346554. doi: 10.1371/journal.pone.0346554 (PMC13739170; doi:10.1371/journal.pone.0346554)
Supplement: Supplementary file 1 [file pone.0346554.s002.pdf]

ScreenIT Report

This is an automated report for 10.64898+2026.03.23.713795.pdf. This report was solicited by the PLOS One editorial team and provided by ScreenIT.

ScreenIT is an independent group of scientists developing automated tools that analyze academic papers. A set of automated tools screened your submitted manuscript and provided the report below. Each tool was created by your academic colleagues with the goal of helping authors.

The tools look for factors that are important for transparency, rigor and reproducibility, and we hope that the report might help you to improve reporting in your manuscript. Within the report you will find links to more information about the items that the tools check. These links include helpful papers, websites, or videos that explain why the item is important.

While our screening tools aim to improve and maintain quality standards they may, on occasion, miss nuances specific to your study type or flag something incorrectly. Each tool has limitations that are described on the ScreenIT website. The tools screen the main file for the paper; they are not able to screen supplements stored in separate files.

Please note that the Academic Editor had access to these comments while making a decision on your manuscript. The Academic Editor may ask that issues flagged in this report be addressed.

If you would like to provide feedback on the ScreenIT tool, please email the team at ScreenIt@bih-charite.de. If you have questions or concerns about the review process, please contact the PLOS One office at plosone@plos.org.

How to read the report:

- no action required

? - review for potential issues

! - a critical issue was found by a tool

n/a - may not be applicable to your paper

- more information about this item can be obtained using this link

Rigor

✓	Ethics	IRB: "Methods Ex vivo testing This study was approved by the Human Research Ethics Committee of the University of New South Wales (HREC number: HC220136)."<br>Field Sample Permit: "The swabs were then put into AMIES transport media (Bacto Laboratories, Australia) and transported to the microbiology laboratory of the School of Optometry and Vision Science University of New South Wales (UNSW) in cold chain and processed within 4 hours of collection."
?	Inclusion and Exclusion Criteria	Not detected. Please specify any pre-defined criteria used to determine whether participants, animals or samples were eligible for the study, or exclude participants, animals or samples from the study.
?	Flow charts and attrition	We did not find a study flow chart of excluded observations. We strongly recommend using flow charts because they provide an overview of the study design and more information about attrition. If you included a study flow chart in your supplemental files, we apologize for missing this. Our tool is not able to screen separate supplemental files.<br><br>Sentence about attrition: not detected. Please provide information about the drop-out of subjects, or loss of animals or samples. This could be done using a study flow chart, or described in the text.
?	Sex as a biological variable	Not detected. Please consider reporting the sex of human participants, animals or cells, if you haven't done so.
?	Subject Demographics	Age: not detected.<br>Weight: not detected.
✓	Randomization	"After use, the sheath was removed, and sterile cotton swabs (Bacto Laboratories, Mount Pritchard, NSW, Australia) were passed twice over a 2-5 cm2 area from the probes including from the top notched, bottom notched, left side of the handle, right side of

		the handle of one probe, grooves (bent areas) and lense areas of other probe (randomly selected) to examine the numbers and types of microbes present on the endoscopes prior to disinfection (Figure 1)."
?	Blinding	Not detected. Please specify whether blinding was used at various phases of the experiment (e.g., blinding of patients, caregivers, outcome assessments, data analysis).
?	Power Analysis	Not detected. Please state how you determined the sample size for the study. If you performed a power or sample size calculation, please provide details.
n/a	Replication	not required.

Generated by Barzooka (flow charts), SciScore (everything else).
SciScore only screens the text of the methods section; we apologize if the tool has missed items reported elsewhere in the paper or supplements.

Resources

Persistent unique identifiers for research resources, RRIDs, can be used to track these key resources though the literature. They are requested or required in many journals, but even if your journal does not require them, we strongly encourage the use of these for the readers of papers who wish to easily track down the key materials like antibodies, software tools, or mice.

Software and Algorithms	"Recovery of Bacillus spores before and with UVC treatment from different sites of probes was compared using t test with Graph Pad Prism 8 version 8.0.2."	? Graph Pad Prism suggested: GraphPad Prism (RRID:SCR_002798. Please make sure this RRID suggestion is accurate before including in your manuscript.

Generated by SciScore.
SciScore only screens the text of the methods section; we apologize if the tool has missed items reported elsewhere in the paper or supplements.

Transparency

?	Open code	If you wrote code to analyze your data, please consider sharing the code in a public repository. This makes it easier for others to reproduce your analyses, and may also aid others seeking to analyze similar datasets.
?	Open data	If permitted, sharing data on a public repository with a persistent identifier (a DOI or accession number) can improve reproducibility and make it easier for other scientists to expand on your work. Papers with open data are cited more often than papers without open data. Some institutions have an expert who can provide advice on data sharing.
✓	Self-acknowledged limitations	"However, certain limitations should be acknowledged for the recent study. Viral pathogens were not evaluated. Additionally, while environmental contamination was recognized, transmission dynamics were not directly investigated. Future research should explore the effectiveness of UVC against viral pathogens. Another, key limitation of some conventional disinfection approaches, where irregular surfaces can compromise microbial inactivation. However, the UV-C LED system combined with UVMESH technology similar to previous study (20), delivers comprehensive 360 UV-C coverage achieving microbial inactivation across complex probe surfaces in the current study. This supports the reliability and reproducibility of the UV-C disinfection system in real-world clinical environments. In conclusion, the findings demonstrate that reusable clinical probes are frequently contaminated with diverse microbial species under routine conditions. UVC treatment achieved consistent and substantial (> 6 log) micr..."
✓	Conflict of interest statement	Thank you for stating any conflicts of interest.
✓	Funding statement	Thank you for reporting your funding source(s), or stating that there was no specific funding for this work.
n/a	Registration statement	A registered protocol makes the planned research project more transparent and can

	protect against bias. Please consider registering future confirmatory studies.

Generated by ODDPub, limitation-recognizer, rtransparent, trial-identifier.

Figures

?	Bar graphs of continuous data	We found bar graphs of continuous data. We recommend replacing these with more informative graphics (e.g. dotplots, box plots, violin plots), as many different datasets can lead to the same bar graph. The actual data may suggest different conclusions from the summary statistics alone. Please see the following resources for more information on the problems with bar graphs (Weissgerber et al 2015) and what to use instead (Weissgerber et al 2019).
✓	No rainbow color maps	Thank you for not using rainbow colormaps. Rainbow color maps distort readers' perception of the data by introducing visual artifacts and are not accessible to colorblind readers.

Generated by JetFighter, Barzooka.
