# Supplementary material for: Inactivation of microorganisms in the complex regions of transvaginal ultrasound probes by a UVC-LED light based disinfection system
Source: PLoS One. 2026 Nov 5;21(11):e0346554. doi: 10.1371/journal.pone.0346554 (PMC13739170; doi:10.1371/journal.pone.0346554)
Supplement: Supplementary file 2 [file pone.0346554.s001.docx]

**Supplementary Table: 1**

| **Collection day** | **Clinical/Examination Room** | | **Disinfectant room** | |
| --- | --- | --- | --- | --- |
|  | **Bacterial type** | **Numbers (log_10_ CFU)** | **Bacterial type** | **Numbers (log_10_ CFU)** |
| *Day 1* | *S. hominis*  *S. warneri*  *S. epidermidis*  *E. faecium*  *M. luteus*  *S. haemolyticus*  *Corynebacterium riegelii* | 2.9  2.8  1.8  1.6  1.6  1.2  1.1 | *S. epidermidis*  *M. luteus*  *S. hominis*  *K. rhizophila*  *S. warneri*  *E. faecium* | 1.6  1.3  1.1  0.9  0.9  0.8 |
| *Day 2* | *K. rhizophila*  *B. cereus*  *S. hominis*  *S. warneri* | 1.9  1.9  1.7  1.5 | *S. hominis*  *S. pasteuri*  *S. haemolyticus*  *P. koreensis*  *S. epidermidis* | 1.1  0.9  0.9  0.6  0.6 |
| *Day 3* | *S. haemolyticus*  *B. cereus*  *S. warneri*  *S. epidermidis*  *Dermacoccus nishinomiyaensis* | 2.1  1.6  1.3  0.9  0.6 | *S. epidermidis*  *S. epidermidis*  *K. rhizophila*  *B. cereus*  *S. capitis*  *Streptococcus sanguinis* | 1.3  1.3  1.2  1  0.9  1.1 |
| *Day 4* | *S. hominis*  *C. aurimucosu*  *S. cohnii*  *L. pneumophila*  *M. luteus*  *B. cereus*  *S. arlettae* | 2.6  2.3  1.9  1.8  1.7  0.9  0.6 | *S. epidermidis*  *Dermatophilus congolensis*  *M. luteus*  *Kocuria rhizophila*  *E. faecium*  *S. condiment*  *S. lugdunensis* | 1.9  1.9  1.6  1.3  1.0  0.8  0.6 |

Numbers of bacteria identified in the environment where probes were sampled and processed.
