# Supplementary material for: Inactivation of microorganisms in the complex regions of transvaginal ultrasound probes by a UVC-LED light based disinfection system
Source: PLoS One. 2026 Nov 5;21(11):e0346554. doi: 10.1371/journal.pone.0346554 (PMC13739170; doi:10.1371/journal.pone.0346554)
Supplement: Supplementary file 3 [file pone.0346554.s004.docx]

**Reviewer #2:**

Dear Authors,
Thank you for submitting your manuscript on the efficacy of a UV-C LED disinfection system for transvaginal ultrasound probes. The study addresses an important issue in infection prevention, and the combination of real-world sampling with controlled spore experiments is a valuable approach.
That said, the manuscript requires significant revision to improve clarity, methodological rigor, and scientific presentation.

**Major comments**
**Manuscript completeness and clarity**
**Comment:** The current version appears incomplete and contains numerous fragmented sentences, missing values, and formatting issues (e.g., missing units, incomplete log reduction values, broken tables, and placeholder text). These issues make it difficult to fully assess the study. Carefully revise the entire manuscript for completeness. Ensure all numerical data (e.g., log reductions, concentrations, volumes) are clearly reported with correct units. Reconstruct tables and figures so they are readable and interpretable.

**Response:** Thank you for your suggestions. We have revised the whole manuscript as suggested. We have reconstructed the figures and tables. Please see Figure 2 and Tables 1 and 2, completed the missing values and units and described volume with units etc.

**Methodological transparency**
Key methodological details are insufficient or unclear:
**Comment:** Clarify the sampling protocol (number of replicates, timing, handling procedures).

**Response:** Thank you for your comment. We have clarified all the requested information’s in the revised manuscript as following. (**See page#05, line 105-123)**

The ultrasound probes were collected (from total thirty subjects) after use in female (aged >18 years) an in vitro fertilization clinic (Connect IVF, Sydney, Australia) from February 26 to April 02, 2025. All the subjects completed the study. These probes had been sheathed with a non-sterile latex free probe cover (Sonologic) prior to use. After use, the sheath was removed, and sterile cotton swabs (Bacto Laboratories, Mount Pritchard, NSW, Australia) were passed twice over a 2-5 cm^2^ area from the probes including from the top notched, bottom notched, left side of the handle, right side of the handle of one probe, grooves (bent areas) and lense areas of other probe (randomly selected) to examine the numbers and types of microbes present on the endoscopes prior to disinfection. The ultrasound probes were then loaded into the Lumicare ONE High Level Disinfection UVC system (Lumicare 606A743U7Y, Sydney, Australia) and the system run for a standard time of 90 seconds. After disinfection, the ultrasound probe was removed, and the opposite side of each probes’ sites was swabbed as described above. The swabs were then put into AMIES transport media (Bacto Laboratories, Australia) and transported to the microbiology laboratory of the School of Optometry and Vision Science University of New South Wales (UNSW) in cold chain and processed within 4 hours of collection.

**Comment:** Provide precise details of the UV-C system (dose, wavelength, distance, exposure uniformity).

**Response:** The Lumicare system is an automated HLD system (the device does everything on its own, dose determination). The previous study demonstrates that the Lumicare UVC-LED system (Lumicare 606A743U7Y, Sydney, Australia) disinfected the ultrasound probes after 90 sec cycle and effectively eliminated microorganisms associated with them.

During a standard HLD cycle, UVC LEDs generate UVC irradiation (265 nm – 275 nm) across the entire surface of the ultrasound probe positioned within the enclosed disinfection chamber at an approximate distance of 3 cm**. (See page#5, line 116-118)**

**Comment:** Explain how contamination rates and “100% decontamination” were defined.

**Response:** We added the following information’s in the revised manuscript **(See page#11, line 203-206)**

1. Rate of contamination of specific site was defined based on the following formula

- Number of site positive for microbial growth/ total number of sites screened for bacterial growth× 100.

2. While 100 % decontamination for specific site was defined based on the following formula

- Number of site negative for microbial growth/ total number of sites screened for bacterial growth× 100.

**Comment:** Describe statistical analysis more clearly (e.g., how P values were calculated with small sample sizes). Sample size and study design limitations.

**Response:** Due to the small sample size, the data were not assessed for normal distribution and were therefore analysed using non-parametric statistical tests to determine the P value. This study also has several general limitations, including sample collection from a single laboratory and the limited number of probes with complex geometries evaluated. These factors constrained both the sample size and the overall study design, which may limit the generalisability of the findings. These details have been added in the revised manuscript. **Please see Page# 8, Line# 179-182.**

**Comment:** The study is based on only two probes, which limits generalizability. This limitation should be explicitly acknowledged and discussed in more depth.

**Response:** We have included the following details in the revised manuscript. Please see **Page#19-20, Line#407-417.**

Another key limitation of this study was the inclusion of only two probes from a single probe category. Ultrasound probes are broadly classified into endocavity probes (e.g., transvaginal and transrectal) and external probes (e.g., linear, curved, and phased array) (1). The present study evaluated the effectiveness of the Lumicare UVC-LED system only against endocavity probes, using representative commonly used transvaginal/transrectal ultrasound probes manufactured by Mindray. However, each probe type contains unique and complex surface geometries that require independent evaluation for effective disinfection. Consequently, this study provides limited insight into the disinfection performance across the broader range of ultrasound probe types. Therefore, future studies should evaluate the effectiveness of the Lumicare UVC system on multiple probe types with varying structural complexities.

**Comment:** Avoid overgeneralizing conclusions to broader clinical settings.

**Response:** Thank you for your suggestion. We have rewritten the conclusion as following. **Please see Page#20, Line#418-422.**

In conclusion, the findings demonstrate that the Lumicare UVC-LED system can effectively disinfect both straight and curved transvaginal probes by inactivating microorganisms residing in complex probe sites such as notches, handle, grooves, and lense areas etc. Furthermore, UVC treatment achieved consistent and substantial (> 6 log₁₀) reduction of B. subtilis spores across all tested complex sites, highlighting its potential to inactivate highly resistant microorganisms.

**Data consistency and interpretation**

**Comment:** There are inconsistencies and unclear reporting in the results:

Log reduction values are incomplete or inconsistently presented (e.g., “>6.7 l”, missing units). Some numerical results appear truncated or misaligned.

**Response:** Thank you for your suggestions. We have added unites with all the values.

**Comment:** Ensure consistency between text, tables, and figures.

**Response:** We have now rewritten everything consistently throughout text, tables, and figures.

**Overstatement of conclusions**
**Comment:** While the results are promising, statements such as “100% decontamination” and “complete elimination” should be interpreted cautiously:

**Response:** Thank you for your suggestions. We have carefully edited the words 100% decontamination. We have also deleted the words “complete elimination” and changed to “effectively elimination” wherever applicable.

Thank you for your suggestion we have changed words “complete elimination” to “decontamination: wherever applicable throughout the revised manuscript.

**Comment:** Discuss detection limits and potential residual contamination. Avoid implying absolute sterility unless this is methodologically justified.

**Response:** We greatly acknowledge your suggestions. We have removed the wordings “detection limits and potential residual contamination” and rewritten the sentence as “The small spore’s counts (0.23–0.33 log₁₀ CFU) may be attributable to the detection of organisms near the assay’s detection limit.”

**Literature context and novelty**
The discussion would benefit from clearer positioning within existing literature:
**Comment:** Expand comparison with previous studies on UV-C disinfection of ultrasound probes.

**Response:** A previous study reported the detection of Human Papillomavirus (HPV) on vaginal ultrasound probes and demonstrated that the virus was completely inactivated by a UVC disinfection system within a 90 sec disinfection cycle (2). **Page #19, Line #403-405**

In a previous study, a UVC device achieved a 2.17 log₁₀ reduction of S. aureus, E. faecalis, and E. coli within 5–7 min when disinfecting probes (3). The longer disinfection time (5–7 min) reported in the previous study, compared with the 90-second cycle in the recent study, may be attributed to probe disinfection being performed in the presence of ultrasound gel. In contrast, the gel was completely removed prior to disinfection in the present study. The presence of gel may have impeded UVC penetration, thereby reducing disinfection efficiency and necessitating a longer exposure time. **Page #17, Line #335-341**

A previous study reported that 3D scanning of endocavity bi-plane and curved array probes demonstrated an uneven distribution of irradiance across probe regions when exposed to a UVC system (4), which may compromise the performance of the disinfectant system. However, the current UVC-LED device incorporating UVMESH technology achieved complete microbial inactivation, including on complex region of both probe types. This difference may be attributed to variations in study design, as the previous study primarily modelled or assumed irradiance distribution across differently designed probe surfaces rather than evaluating the real-world antimicrobial efficacy of the disinfection system. In contrast, the present study directly assessed microbial inactivation on probe surfaces under practical conditions. Additionally, probe design and geometry can also contribute to variations in irradiance distribution across specific probe sites (4). **Page #16, Line #314-324.**

**Comment:** Clarify what is novel about your system (e.g., UVMESH technology) beyond existing UV-C approaches.

**Response:** Thank you for asking clarification on UVMESH technology. We have added following paragraph in the revised manuscript. Please see **Page#16, Line # 324-329.**

This suggests that UVC-LEDs delivered UVC light effectively across the entire probe surface using UVMESH technology, resulting in the elimination of microorganisms from complex and uneven probe surfaces (5). This technology enhances spatial distribution and more uniform irradiance on entire area of target surfaces. Moreover, this approach specifically minimises the risk of small areas remaining untreated and maximises decontamination efficiency, even across complex surface architectures and geometries. Additionally, the system is engineered to provide controlled and reproducible UVC exposure conditions, potentially enhancing consistency in microbial inactivation outcomes.

**Language and structure**
The manuscript requires substantial language editing:
**Comment:** Improve grammar, sentence structure, and readability.

**Response:** The revised manuscript has been carefully edited for grammar and improved for readability.
**Comment:** Remove repetition and ensure logical flow between sections.

**Response:** All repetitions have been removed, and the sentences are now coherent.

**Comment:** Ensure consistent terminology (e.g., UVC vs. UV-C, probe regions).

**Response:** We have consistently used UVC by replacing UV-C and regions by sites in the body text of whole manuscript.  **Minor comments**
**Comment:** Clearly define all abbreviations at first use.

**Response:** The abbreviations when introduced first time in the manuscript are defined. Please see as below

1. Colony forming units (CFU)
2. Matrix-Assisted Laser Desorption/Ionization Time-of-Flight (MALDI-TOF)
3. α-cyano-4-hydroxycinnamic acid (HCCA)
4. Chocolate Blood agar (CBA)
5. Saboraud dextrose agar (SDA)
6. Phosphate Buffer Saline (PBS)

**Comment:** Standardize bacterial nomenclature (italicization, full names).

**Response:** All the bacterial names written with full name in the manuscript, have been italicized.
**Comment:** Ensure all references are properly formatted and complete.

**Response:** All the references have been reformatted according to the journal requirements.

**Comment:** Improve figure captions to be self-explanatory.

**Response:** We have rewritten the figure captions for better understanding. Please see Figure 2 caption.

**Figure 2.** Effect of UVC treatment on recovery of *B. subtilis* spores from different sites of probes. Spores’s viable counts (log₁₀ CFU/site) recovered from control (non UVC treated) and samples (UVC treated) are shown for the handle, bottom notch and lens (**Probe A)**, and central bent side, left side of edge and lens of **(Probe B**). Recovery of spores from control sites were > 6.5 log₁₀ CFU/site, however after UVC treatment recovery of spores from samples sites decreased to < 0.5 log₁₀ CFU/site. This shows that UVC-LED system reduced > 6 log₁₀ bacillus spores on complex sites of both probes.

**References**

The following references were cited in support of our response.

1.	Shokoohi H, Armstrong P, Tansek R. Emergency department ultrasound probe infection control: challenges and solutions. *Open Access Emerg Med*. 2015;7:1-9.

2.	Pichon M, Lebail-Carval K, Billaud G, Lina B, Gaucherand P, Mekki Y. Decontamination of intravaginal probes infected by human *papillomaviru*s (HPV) using UVC decontamination system. *J of Clinic Med*. 2019;8(11):1776.

3.	Bloc S, Mercadal L, Garnier T, Komly B, Leclerc P, Morel B, et al. Evaluation of a new disinfection method for ultrasound probes used for regional anesthesia: ultraviolet C light. *J Ultrasound Med*. 2011;30(6):785-8.

4.	Yeoh L, Cogar L, Barak M, Tan LY, Spargo G, Burdach J. UV-C disinfection of ultrasound probes: Challenges of uneven irradiance on complex surfaces. *PLoS One*. 2024;19(10):e0312931.

5.	<https://lumicare.one/uvmesh>. (Accessed on 02/06/2026) [
