# Supplementary material for: Inactivation of microorganisms in the complex regions of transvaginal ultrasound probes by a UVC-LED light based disinfection system
Source: PLoS One. 2026 Nov 5;21(11):e0346554. doi: 10.1371/journal.pone.0346554 (PMC13739170; doi:10.1371/journal.pone.0346554)
Supplement: Supplementary file 4 [file pone.0346554.s005.docx]

**Comments to the Author**

Please use the space provided to explain your answers to the questions above. You may also include additional comments for the author, including concerns about dual publication, research ethics, or publication ethics. (Please upload your review as an attachment if it exceeds 20,000 characters)

**Reviewer #3:**

In Manuscript PONE-D-26-13842_R1, Yasir and Willcox present their research on disinfection of transvaginal ultrasound probes with a UVC-LED based system. Overall, the revision presents solid data on this topic, but the manuscript lacks a certain flow with respect to the presentation of their data. In its current form, the detailed data presented in the Results section often makes reading the manuscript difficult. Overall, the paper is straightforward, it is more confusing than it needs to be.

- Transvaginal probes are dirty and become contaminated with routine use
- Clinical and disinfection rooms are dirty and may contribute to contamination
- Current cleaning methods are ineffective (however, there is no proof of this in the current data, it is implied)
- A new UVC disinfection method can significantly reduce contamination as shown by P in vitro and ex vivo testing

I have put suggestions below:

**Comment:** Each section of the Results (e.g., Ex vivo testing), the reason behind the study is not presented. For example, On Page 22 Line 191, the section starts with a statement that “areas of probes A and B were heavily contaminated with microbes…” A statement such as, “To demonstrate the level of contamination on transvaginal ultrasound probe, various areas of the probes were swabbed and examined for the presence of bacteria.”

Each section would benefit by walking the reader through the flow of the experiments.

**Response:** Thank you for your suggestions. We have added similar in each section wherever required. Such as;

1. To demonstrate the level of contamination on transvaginal ultrasound probe, various areas of the probes were swabbed and examined for the presence of bacteria.
2. To confirm that each probe was effectively disinfected prior to spores’ inoculation before each experimental repeat, 4% bleach was used.
3. In order to determine the efficacy of UVC treatment, bleach (4%) sterilized probes were inoculated with control bacillus spores and the reduction in recovered spores were measured before and after UVC treatment.

**Comment:** The detailed analysis of contamination by various bacterial species (page 23 Line 195-206) is hard to read. Do the authors believe that the different notches, sides of the handle etc., are contaminated differently because how they are used? Or are these areas just difficult to clean portions of the probe? It is fine to list the details about log10CFU for different species in the table, but the paragraph is hard to read. I think that just mentioning the variety of species found and the range of contamination (log10CFU) in Table 1 would be sufficient.
Giving detailed species for different regions of the probes suggests that the design is important. I believe that the authors are attempting to demonstrate the high level of contamination before disinfection and the variety of species. The level of detail presented would suggest there is more to the story.

**Response:** Thank you for giving your valuable suggestions.

- We have adjusted the paragraph as suggested. We have removed the information’s from paragraph and retained in the Table 1.
- Different regions of the probes such as notches, groves, sides of the handle are difficult to clean areas of the probes. Therefore, we have chosen to determine the disinfectant performance of UVC-LED system against the microorganisms recovered from these complex and difficult to clean areas. **(please see page# 15, Line#329-330)**
- We have updated this information about (the range of contamination (log10CFU)) in the revised manuscript. **(please see page# 9, Line#215 and 220)**

**Comment:** The authors describe the rate of contamination of various portions of the probes (Page 25 Line 209-222) and this section is much easier to read/follow. Similarly, a simpler presentation of the data mentioned above in Comment 2, would improve the paper.

**Response:** Thank you for your suggestions. As suggested, we have rewritten and shortened the paragraph.

**Comment:** Page 26 Line 238 This section could also use an introductory statement, such as “probe covers are often utilized to prevent contamination of probes, but they could also serve as a source of infections.” Similarly, remove the log_10_ CFU in the written paragraph and refer readers to the table for those values. Again, contamination is contamination but do the different species matter or is again just demonstration of the level of contamination.

Probe covers were examined on the inside so again the level of contamination is impressive but would be expected.

**Response:** As suggested, we have added

1. “probe covers are often utilized to prevent contamination of probes, but they could also serve as a source of infections.” We have removed log10 CFU values written in the paragraph and wrote “refer to Table 3” in the paragraph. **(please see page# 12, Line#253 and 254)**
2. Yes, agree with your opinion that contamination is contamination either one or many bacteria and with single CFU or many CFUs. However, we have described bacterial contamination, including the identification of species and their respective levels to maintain consistency throughout the manuscript and to provide a comprehensive representation of the microbial profiles observed across all data obtained in this study.
3. The contamination of probe cover is expected. In previous study Yasir and Willcox (1), we have determined that probe covers from inside was contaminated with *Bacillus subtilis*.

We have included following line in the revised manuscript to support our statement.

4. Previous study demonstrated that probe covers were contaminated with *Bacillus subtilis* with level up to 2.8 log10 CFU (1). **(please see page# 18, Line#398 and 399)**

**Comment:** For the probes/covers examined with ex vivo testing, it appears that they are heavily contaminated. How were they cleaned prior to use? Were they cleaned using standard protocols? Did the authors demonstrate that they were clean prior to use? In other words, does this contamination represent the “cleaned” probes that were assumed to be cleaned by current methods (non-UVC)? If so, then the authors need to comment on the quality of current sterilization methods, because this is the impetus for the need for a new sterilization method or UVC.

**Response:** Thank you for seeking clarification. Please find our response below.

- Prior to in vivo use in the clinic, probes were sheathed with non-sterile, latex-free probe covers, and coated with non-sterile gel. Also no wipe-down or manual cleaning step was performed between removal of the sheath and swab sampling — this was a deliberate study design choice to capture worst-case contamination. **(please see page# 6, Line#138 and 139)**
- Yes, this is the key methodological point in this study we deliberately followed the standard procedures undertaken within an Ultrasound department prior to HLD. This allowed us to test the UVC system under standard real-world conditions. In the standard protocol the probes were wiped over with a paper towel to remove debris.
- In routine clinical practice, ultrasound probes are cleaned with paper towel or wipes to remove visible contamination, including debris and organic material. However, these cleaning methods may not achieve complete removal or inactivation of microorganisms on the probe surface. Therefore, for semi-critical devices, HLD is required following the cleaning step as part of standard infection prevention and control protocols.

**Comment:** On Page 21 Line 168-9, the authors state that probes were disinfected with 4% ethanol. Is this the standard sterilization process? The authors need to give the reader more background about standard sterilization. If current methods are failing or had been assumed to be adequate, then again, the need for a new disinfection method is obvious. The authors are missing opportunities to make their current work relevant and more importantly necessary for disinfection.

**Response:** Our response is below.

- We apologise for this error. In the in vitro experiments, the probes were treated with 4% sodium hypochlorite (bleach) to achieve repeated sterility between experimental runs. This procedure was used specifically to ensure that the probes were free of viable microorganisms before subsequent inoculation with spores. We acknowledge that treatment with 4% sodium hypochlorite is not a standard sterilisation method for clinical ultrasound probes and was used solely as part of the experimental protocol for the in vitro study. However, this is not recommended by probe manufacturers due to the potential for damage to the metal and plastic components of the probes (2). **(See page# 18, Line#410-412).**
- Various methods, including chemical soaking, chemical aerosolisation, and surface wiping, have been employed for ultrasound probe disinfection. Chemical soaking requires the probe to be immersed in a disinfectant solution for approximately 8–45 minutes, whereas chemical aerosolisation involves nebulisation of hydrogen peroxide within a device chamber and may require approximately 7–12 minutes, depending on the system and operating conditions. Quaternary ammonium compounds and chlorhexidine in solution (3), or soaked with wipes (4) found ineffective against HPV. Similarly, glutaraldehyde exhibits broad-spectrum antimicrobial activity, but its use is limited due to potential damage to ultrasound probes (5). These chemical-based approaches may present occupational and environmental concerns due to potential exposure to chemical disinfectants. **(See page# 4, Line#83- 93).**

**Comment:** Is there any published data about cross-infection between patients following the use of a transvaginal ultrasound probe? Yes, probes are dirty following their use, but what does it look like after standard cleaning? The paper would be enhanced if the authors had compared standard disinfection with the new UVC based method. I am not asking for more experiments to be done, but if this data is available, then the paper would be greatly strengthened in relevance and importance.

**Response:**

1. The real risk of infection associated with TVUS probes following use without adequate decontamination is unknown. No case of related specific infection (cross-contamination between patients) has been reported in the literature, but contaminated ultrasound transducers can be potential vector for transfer of microorganisms.
2. The cleaning process, which precedes disinfection, is crucial in removing organic matter that can interfere with the effectiveness of disinfectants. Inadequate cleaning can result in residual contaminants that provide a protective barrier for pathogens, rendering disinfection ineffective. This scenario can lead to the transmission of infections, posing serious risks to patients.
3. According to the Australasian Society for Ultrasound in Medicine (ASUM) and Australian Standard (AS5369), American Institute of Ultrasound in Medicine (AIUM) guidelines, transvaginal ultrasound probes are classified as semi-critical devices and require high-level disinfection (HLD) following cleaning. Various disinfectants have been used for probe disinfection, including quaternary ammonium compounds, chlorhexidine, and quaternary ammonium-based wipes. Although these agents are effective against a range of bacteria and fungi within 5-20 minutes, their effectiveness against certain viruses, including HPV, may be limited (3, 4). Sherman et al. reported that 70% alcohol was highly effective against bacterial contamination but demonstrated limited activity against viral contamination. Similarly, glutaraldehyde, ortho-phthalaldehyde, and 4% chlorhexidine gluconate were found to be ineffective against HPV16 (6, 7). However, UV-C devices can disinfect vaginal ultrasound probes faster and can inactivate all the microorganism including viruses (8, 9). **(See page# 16, Line#344-355)**

In addition, chemical disinfectants may also damage to surface of ultrasound probe. Although sodium hypochlorite has broad-spectrum antimicrobial activity, its potential for surface damage limits its use for probe disinfection (2). In contrast, independent testing demonstrated that repeated exposure to the Lumicare UV-C LED system did not adversely affect probe surface. **(See page# 18, Line#410-412) and (See page#19 , Line#428-431).**

**Comment:** The authors should include a figure of the UVC-LED based system for completeness.

**Response:** Thank you for your suggestions. We have added diagram UVC Lumicare device. (See Figure 1)

**Figure: 2.** Lumicare UVC-LED based system: frontal view (A) and chamber view showing the probes suspended inside the chamber (B).

**Comment:** For the In vitro testing section (page 28 Line 271-291), again an opening introductory line describe the experiment would help the flow of the Results section. For example, “In order to determine the efficacy of UVC treatment, bleach sterilized probes were inoculated with control bacteria and the reduction in recovered bacteria were measured before and after UVC treatment.”
Again, does specific efficacy of UVC treatment on various sites (e.g., lens, central bent side etc.) matter? The authors are not attempting to comment on the design of the probe, just that certain regions with 3D structures are more prone to contamination. I am not sure such details matter for the study and could be shortened, removed, or the range of log10 reductions could be presented.

**Response:** Thank you for your suggestions we have added the following statement.

1. “In order to determine the efficacy of UVC treatment, bleach sterilized probes were inoculated with control bacteria and the reduction in recovered bacteria were measured before and after UVC treatment.” **(Please see Page #13, Line# 280-282)**
2. We have discussed and commented on the design of the probe in discussion section **(Please see Page #20, Line# 452-461)**
3. All authors agreed to retain the detailed Log₁₀ values for each site of the probe in the Results section of the revised manuscript.

**Comment:** The analysis of baseline contamination of the clinical and disinfectant rooms is relevant to the study, but why was air sampling not used instead? Open agar plates were left in the room, but a more complete analysis of room contamination is often done with an SAS 180 Impaction Air Sampler. Again, not asking for more experiments at this stage of submission, but for future studies, I would suggest use of such a device.
I would move this section to either the end of Results or I would consider adding it as a supplemental table.

**Response:**

1. As suggested, we have removed this table from main part of manuscript and added as Supplementary Table 1.
2. The air sampler will be used in future studies to investigate the relationship between environmental microbial contamination and ultrasound probe contamination.

We have also included the following statement in the revised manuscript.

3. While environmental contamination was documented but its transmission dynamics was not determined, future research should investigate the transmission dynamics with an SAS 180 Impaction Air Sampler**. (Please see Page #19-20, Line#440-442)**

**Comment:** In the Discussion (page 32 Line 374-390), the authors discuss at length the various types of bacteria found on probes and in the rooms. The detailed discussion about different types of bacteria common to both sites is of interest, but not as much as their results on cleaning the probes/disinfection system. Since there was no proof of cross-contamination between patients, this section is purely hypothetical and doesn’t add to their major findings.

**Response:** Thank you for your suggestions. We have reduced the details in this paragraph **(Please see Page #19, Line#436-440).**

**References**

1.	Yasir M, Willcox MDP. The use of ultraviolet light generated from light-emitting diodes for the disinfection of transvaginal ultrasound probes. *PLoS One*. 2024;19(2):e0298449.

2.	Abramowicz JS, Evans DH, Fowlkes JB, Maršal K, terHaar G. Guidelines for Cleaning Transvaginal Ultrasound Transducers Between Patients. *Ultra in Med & Biol*. 2017;43(5):1076-9.

3.	M'Zali F, Bounizra C, Leroy S, Mekki Y, Quentin-Noury C, Kann M. Persistence of microbial contamination on transvaginal ultrasound probes despite low-level disinfection procedure. *PloS one*. 2014;9(4):e93368.

4.	Casalegno J-s, Le Bail Carval K, Eibach D, Valdeyron M-L, Lamblin G, Jacquemoud H, et al. High risk HPV contamination of endocavity vaginal ultrasound probes: an underestimated route of nosocomial infection? *PloS one*. 2012;7(10):e48137.

5.	Zambrano-Carrillo MF, Hernandez-Jerez AF. Occupational risk assessment of glutaraldehyde through personal air monitoring in a hospital setting. *Ann of Work Expos and Health*. 2024;68(1):97-103.

6.	Meyers J, Ryndock E, Conway MJ, Meyers C, Robison R. Susceptibility of high-risk human papillomavirus type 16 to clinical disinfectants. *J Antimicrob Chemother*. 2014;69(6):1546-50.

7.	Ienghong K, Kleebbuakwan K, Apiratwarakul K, Phungoen P, Gaysonsiri D, Bhudhisawasdi V. Comparison of Cleaning Methods for Ultrasound Probes at an Emergency Department in a Resource-Limited Country. *J of the Med Asso of Thai*. 2020;103:67.

8.	Meyers C, Milici J, Robison R. UVC radiation as an effective disinfectant method to inactivate human papillomaviruses. *PLoS One*. 2017;12(10):e0187377.

9.	Pichon M, Lebail-Carval K, Billaud G, Lina B, Gaucherand P, Mekki Y. Decontamination of intravaginal probes infected by human *papillomaviru*s (HPV) using UVC decontamination system. *J of Clinic Med*. 2019;8(11):1776.
