# Supplementary material for: Inactivation of microorganisms in the complex regions of transvaginal ultrasound probes by a UVC-LED light based disinfection system
Source: PLoS One. 2026 Nov 5;21(11):e0346554. doi: 10.1371/journal.pone.0346554 (PMC13739170; doi:10.1371/journal.pone.0346554)
Supplement: Peer review history

# Peer review: Decision Letter 0

**DOI:** 10.1371/journal.pone.0346554.r001

**Authors:** Marcos Pileggi

28 Apr 2026

Dear Dr. Yasir,

Thank you for submitting your manuscript to PLOS ONE. After careful consideration, we feel that it has merit but does not fully meet PLOS ONE’s publication criteria as it currently stands. Therefore, we invite you to submit a revised version of the manuscript that addresses the points raised during the review process.

Despite the significant scientific contributions of this study, deficiencies in spelling and formatting have impeded the clarity of the text. Furthermore, concerns regarding the interpretation of the results warrant careful attention by the authors to facilitate the continuation of the publication process.

Please submit your revised manuscript by Jun 11 2026 11:59PM. If you will need more time than this to complete your revisions, please reply to this message or contact the journal office at plosone@plos.org. When you're ready to submit your revision, log on to https://www.editorialmanager.com/pone/ and select the 'Submissions Needing Revision' folder to locate your manuscript file.

- A letter that responds to each point raised by the academic editor and reviewer(s). You should upload this letter as a separate file labeled 'Response to Reviewers'.
- A marked-up copy of your manuscript that highlights changes made to the original version. You should upload this as a separate file labeled 'Revised Manuscript with Track Changes'.
- An unmarked version of your revised paper without tracked changes. You should upload this as a separate file labeled 'Manuscript'.

If you would like to make changes to your financial disclosure, please include your updated statement in your cover letter. Guidelines for resubmitting your figure files are available below the reviewer comments at the end of this letter.

If applicable, we recommend that you deposit your laboratory protocols in protocols.io to enhance the reproducibility of your results. Protocols.io assigns your protocol its own identifier (DOI) so that it can be cited independently in the future. For instructions see: https://journals.plos.org/plosone/s/submission-guidelines#loc-laboratory-protocols. Additionally, PLOS ONE offers an option for publishing peer-reviewed Lab Protocol articles, which describe protocols hosted on protocols.io. Read more information on sharing protocols at https://plos.org/protocols?utm_medium=editorial-email&utm_source=authorletters&utm_campaign=protocols.

As the corresponding author, your ORCID iD is verified in the submission system and will appear in the published article. PLOS supports the use of ORCID, and we encourage all coauthors to register for an ORCID iD and use it as well. Please encourage your coauthors to verify their ORCID iD within the submission system before final acceptance, as unverified ORCID iDs will not appear in the published article. *Only*  the individual author can complete the verification step; PLOS staff *cannot*  verify ORCID iDs on behalf of authors.

We look forward to receiving your revised manuscript.

Kind regards,

Marcos Pileggi, Ph.D

Academic Editor

PLOS One

Journal requirements:

When submitting your revision, we need you to address these additional requirements.

1. Please ensure that your manuscript meets PLOS ONE's style requirements, including those for file naming. The PLOS ONE style templates can be found at

https://journals.plos.org/plosone/s/file?id=wjVg/PLOSOne_formatting_sample_main_body.pdf and

https://journals.plos.org/plosone/s/file?id=ba62/PLOSOne_formatting_sample_title_authors_affiliations.pdf

2. Please provide additional details regarding participant consent. In the ethics statement in the Methods and online submission information, please ensure that you have specified (1) whether consent was informed and (2) what type you obtained (for instance, written or verbal, and if verbal, how it was documented and witnessed). If your study included minors, state whether you obtained consent from parents or guardians. If the need for consent was waived by the ethics committee, please include this information.

If you are reporting a retrospective study of medical records or archived samples, please ensure that you have discussed whether all data were fully anonymized before you accessed them and/or whether the IRB or ethics committee waived the requirement for informed consent. If patients provided informed written consent to have data from their medical records used in research, please include this information.

3. Please ensure that you refer to Figures 1 and 2 in your text as, if accepted, production will need this reference to link the reader to the figure.

If the reviewer comments include a recommendation to cite specific previously published works, please review and evaluate these publications to determine whether they are relevant and should be cited. There is no requirement to cite these works unless the editor has indicated otherwise.

[Note: HTML markup is below. Please do not edit.]

Reviewers' comments:

Reviewer's Responses to Questions

**Comments to the Author**

1. Is the manuscript technically sound, and do the data support the conclusions?

Reviewer #1: Yes

Reviewer #2: Yes

**********

2. Has the statistical analysis been performed appropriately and rigorously?

Reviewer #1: Yes

Reviewer #2: No

**********

3. Have the authors made all data underlying the findings in their manuscript fully available?

The PLOS Data policy

Reviewer #1: Yes

Reviewer #2: Yes

**********

4. Is the manuscript presented in an intelligible fashion and written in standard English?

Reviewer #1: Yes

Reviewer #2: No

**********

Reviewer #1: This is an automated report for PONE-D-26-13842. This report was solicited by the PLOS One editorial team and provided by ScreenIT.

ScreenIT is an independent group of scientists developing automated tools that analyze academic papers. A set of automated tools screened your submitted manuscript and provided the report below. Each tool was created by your academic colleagues with the goal of helping authors. The tools look for factors that are important for transparency, rigor and reproducibility, and we hope that the report might help you to improve reporting in your manuscript. Within the report you will find links to more information about the items that the tools check. These links include helpful papers, websites, or videos that explain why the item is important. While our screening tools aim to improve and maintain quality standards they may, on occasion, miss nuances specific to your study type or flag something incorrectly. Each tool has limitations that are described on the ScreenIT website. The tools screen the main file for the paper; they are not able to screen supplements stored in separate files. Please note that the Academic Editor had access to these comments while making a decision on your manuscript. The Academic Editor may ask that issues flagged in this report be addressed. If you would like to provide feedback on the ScreenIT tool, please email the team at ScreenIt@bih-charite.de. If you have questions or concerns about the review process, please contact the PLOS One office at plosone@plos.org.

Reviewer #2: Dear Authors,

Thank you for submitting your manuscript on the efficacy of a UV-C LED disinfection system for transvaginal ultrasound probes. The study addresses an important issue in infection prevention, and the combination of real-world sampling with controlled spore experiments is a valuable approach.

That said, the manuscript requires significant revision to improve clarity, methodological rigor, and scientific presentation.

Major comments

Manuscript completeness and clarity

The current version appears incomplete and contains numerous fragmented sentences, missing values, and formatting issues (e.g., missing units, incomplete log reduction values, broken tables, and placeholder text). These issues make it difficult to fully assess the study.

Carefully revise the entire manuscript for completeness.

Ensure all numerical data (e.g., log reductions, concentrations, volumes) are clearly reported with correct units.

Reconstruct tables and figures so they are readable and interpretable.

Methodological transparency

Key methodological details are insufficient or unclear:

Clarify the sampling protocol (number of replicates, timing, handling procedures).

Provide precise details of the UV-C system (dose, wavelength, distance, exposure uniformity).

Explain how contamination rates and “100% decontamination” were defined.

Describe statistical analysis more clearly (e.g., how P values were calculated with small sample sizes).

Sample size and study design limitations

The study is based on only two probes, which limits generalizability.

This limitation should be explicitly acknowledged and discussed in more depth.

Avoid overgeneralizing conclusions to broader clinical settings.

Data consistency and interpretation

There are inconsistencies and unclear reporting in the results:

Log reduction values are incomplete or inconsistently presented (e.g., “>6.7 l”, missing units).

Some numerical results appear truncated or misaligned.

Ensure consistency between text, tables, and figures.

Overstatement of conclusions

While the results are promising, statements such as “100% decontamination” and “complete elimination” should be interpreted cautiously:

Discuss detection limits and potential residual contamination.

Avoid implying absolute sterility unless this is methodologically justified.

Literature context and novelty

The discussion would benefit from clearer positioning within existing literature:

Expand comparison with previous studies on UV-C disinfection of ultrasound probes.

Clarify what is novel about your system (e.g., UVMESH technology) beyond existing UV-C approaches.

Language and structure

The manuscript requires substantial language editing:

Improve grammar, sentence structure, and readability.

Remove repetition and ensure logical flow between sections.

Ensure consistent terminology (e.g., UVC vs. UV-C, probe regions).

Minor comments

Clearly define all abbreviations at first use.

Standardize bacterial nomenclature (italicization, full names).

Ensure all references are properly formatted and complete.

Improve figure captions to be self-explanatory.

Conclusion

This study has the potential to contribute meaningfully to infection control practices, but substantial revision is needed. Addressing the issues above—particularly manuscript completeness, methodological clarity, and data consistency—will greatly strengthen the work.

I hope these comments are helpful in revising your manuscript.

**********

what does this mean?). If published, this will include your full peer review and any attached files.

If you choose “no”, your identity will remain anonymous but your review may still be made public.

**Do you want your identity to be public for this peer review?**  For information about this choice, including consent withdrawal, please see our Privacy Policy

Reviewer #1: No

Reviewer #2: **Yes:**  Sorana Maria Bucur

**********

[NOTE: If reviewer comments were submitted as an attachment file, they will be attached to this email and accessible via the submission site. Please log into your account, locate the manuscript record, and check for the action link "View Attachments". If this link does not appear, there are no attachment files.]

To ensure your figures meet our technical requirements, please review our figure guidelines: https://journals.plos.org/plosone/s/figures

You may also use PLOS’s free figure tool, NAAS, to help you prepare publication quality figures: https://journals.plos.org/plosone/s/figures#loc-tools-for-figure-preparation.

NAAS will assess whether your figures meet our technical requirements by comparing each figure against our figure specifications.

Attachment Submitted filename: report_10.64898+2026.03.23.713795.pdf

# Author comment: Author response to Decision Letter 1

**DOI:** 10.1371/journal.pone.0346554.r002

14 Jun 2026

Reviewer #1:

This is an automated report for PONE-D-26-13842. This report was solicited by the PLOS One editorial team and provided by ScreenIT. ScreenIT is an independent group of scientists developing automated tools that analyze academic papers. A set of automated tools screened your submitted manuscript and provided the report below. Each tool was created by your academic colleagues with the goal of helping authors. The tools look for factors that are important for transparency, rigor and reproducibility, and we hope that the report might help you to improve reporting in your manuscript. Within the report you will find links to more information about the items that the tools check. These links include helpful papers, websites, or videos that explain why the item is important. While our screening tools aim to improve and maintain quality standards they may, on occasion, miss nuances specific to your study type or flag something incorrectly. Each tool has limitations that are described on the ScreenIT website. The tools screen the main file for the paper; they are not able to screen supplements stored in separate files. Please note that the Academic Editor had access to these comments while making a decision on your manuscript. The Academic Editor may ask that issues flagged in this report be addressed. If you would like to provide feedback on the ScreenIT tool, please email the team at ScreenIt@bih-charite.de. If you have questions or concerns about the review process, please contact the PLOS One office at plosone@plos.org.

Ethics

IRB: "Methods Ex vivo testing This study was approved by the Human Research Ethics Committee of the University of New South Wales (HREC number: HC220136)." Field Sample Permit: "The swabs were then put into AMIES transport media (Bacto Laboratories, Australia) and transported to the microbiology laboratory of the School of Optometry and Vision Science University of New South Wales (UNSW) in cold chain and

processed within 4 hours of collection."

Response: No action required. Thank YOU!

Inclusion and Exclusion Criteria

Not detected. Please specify any pre-defined criteria used to determine whether participants, animals or samples were eligible for the study, or exclude participants, animals or samples from the study.

Response: Sorry for not including the Inclusion and Exclusion criteria in the manuscript. We have now included the Inclusion and Exclusion criteria in the revised manuscript. Please see page# 5, line#98-104.

Inclusion/Exclusion Criteria

Before you decide to participate in this research study, we need to ensure that it is ok for you to take part. The research study is looking recruit people who meet the following criteria:

• 18 years of age or older

• Attending Connect IVF for a transvaginal ultrasound test

• No known or suspected vaginal infections

Participants who meet the following criteria will be excluded from the study:

• Under the age of 18 years

• People highly dependent on medical care who may be unable to give consent

• People with cognitive impairment, an intellectual disability, or a mental illness

Flow charts and attrition

• We did not find a study flow chart of excluded observations. We strongly recommend

using flow charts because they provide an overview of the study design and more

information about attrition. If you included a study flow chart in your supplemental files, we

apologize for missing this. Our tool is not able to screen separate supplemental files.

• Sentence about attrition: not detected.

Please provide information about the drop-out of subjects, or loss of animals or samples. This could be done using a study flow chart or described in the text.

Response: Total thirty subjects were included in the study. All the subject completed the study. These sentences have been added in the revised manuscript. There is no attrition. Therefore, we did not produce flow chart.

Sex as a biological variable

Not detected. Please consider reporting the sex of human participants, animals or cells, if

you haven’t done so.

Response: We have added sex as “female” in the revised manuscript.

Subject Demographics

Age: not detected.

Weight: not detected.

Response: We have added “aged as >18 years” in the revised manuscript

Randomization

"After use, the sheath was removed, and sterile cotton swabs (Bacto Laboratories, Mount Pritchard, NSW, Australia) were passed twice over a 2-5 cm2 area from the probes including from the top notched, bottom notched, left side of the handle, right side of the handle of one probe, grooves (bent areas) and lense areas of other probe (randomly selected) to examine the numbers and types of microbes present on the endoscopes prior to disinfection (Figure 1)."

Response: No response is required. THNAK YOU!

Blinding

Not detected. Please specify whether blinding was used at various phases of the experiment (e.g., blinding of patients, caregivers, outcome assessments, data analysis).

Response: There was no blindness used in the study. Only patients’ numbers were given abbreviation for tracking back to the patients.

Power Analysis

Not detected. Please state how you determined the sample size for the study. If you

performed a power or sample size calculation, please provide details.

Response: Sample sizes were determined based on our previous study;

(Yasir M, Willcox MDP. The use of ultraviolet light generated from light-emitting diodes for the disinfection of transvaginal ultrasound probes. PLoS One. 2024;19(2):e0298449). In previously published study by Yasir et al (2024) included 30 patients, with microbial growth detected in 15 (50%) participants, resulting in statistically significant outcomes. Accordingly, a comparable sample size was adopted in the present study.”

Replication

Response: Not required.

Resources

Software and Algorithms

"Recovery of Bacillus spores before and with UVC treatment From different sites of probes was compared using t test with Graph Pad Prism 8, version 8.0.2.

Graph Pad Prism suggested: GraphPad Prism (RRID:SCR_002798. Please make sure this RRID suggestion is accurate before including in your manuscript.

Response: We have used updated version (11.0.2 (92) of Grpahpad prism to analyse the data. According to GraphPad Prism (RRID:SCR_002798. Graph Pad Prism version 11.0.2.(92) is an appropriate tool to analyse such type of data.

Transparency

Open code

If you wrote code to analyze your data, please consider sharing the code in a public repository. This makes it easier for others to reproduce your analyses, and may also aid

others seeking to analyze similar datasets.

Response: No data has been analysed using code.

Open data

If permitted, sharing data on a public repository with a persistent identifier (a DOI or

accession number) can improve reproducibility and make it easier for other scientists to expand on your work. Papers with open data are cited more often than papers without open data. Some institutions have an expert who can provide advice on data sharing.

Response: All the data is presented in the manuscript. There is no other data to be revealed.

Self-acknowledged limitations

"However, certain limitations should be acknowledged for the recent study. Viral pathogens were not evaluated. Additionally, while environmental contamination was recognized, transmission dynamics were not directly investigated. Future research should explore the effectiveness of UVC against viral pathogens. Another, key limitation of some conventional disinfection approaches, where irregular surfaces can compromise microbial inactivation. However, the UV-C LED system combined with UVMESH technology similar to previous study (20), delivers comprehensive 360 UV-C coverage achieving microbial inactivation across complex probe surfaces in the current study. This supports the reliability and reproducibility of the UV-C disinfection system in real-world clinical environments. In conclusion, the findings demonstrate that reusable clinical probes are frequently contaminated with diverse microbial species under routine conditions. UVC treatment achieved consistent and substantial (> 6 log) micr..."

Response: No response required. Thank YOU!

Conflict of interest statement

Thank you for stating any conflicts of interest.

Response: No response required. Thank YOU!

Funding statement

Thank you for reporting your funding source(s), or stating that there was no specific funding for this work.

Response: No response required. Thank YOU!

Registration statement

A registered protocol makes the planned research project more transparent and can protect against bias. Please consider registering future confirmatory studies.

Response: No response required. Thank YOU!

Figures

Bar graphs of continuous data

We found bar graphs of continuous data. We recommend replacing these with more informative graphics (e.g. dotplots, box plots, violin plots), as many different datasets can lead to the same bar graph. The actual data may suggest different conclusions from the summary statistics alone.

Please see the following resources for more information on the problems with bar graphs (Weissgerber et al 2015) and what to use instead (Weissgerber et al 2019).

Response: Thank you for your suggestions. We have changed our graph to box plots for presenting the data in a better way.

No rainbow color maps

Thank you for not using rainbow colormaps. Rainbow color maps distort readers’ perception of the data by introducing visual artifacts and are not accessible to color blind readers.

Response: No response required. Thank YOU!

References

The following references were cited in support our response.

Yasir M, Willcox MDP. The use of ultraviolet light generated from light-emitting diodes for the disinfection of transvaginal ultrasound probes. PLoS One. 2024;19(2):e0298449.

Reviewer #2:

Dear Authors,

Thank you for submitting your manuscript on the efficacy of a UV-C LED disinfection system for transvaginal ultrasound probes. The study addresses an important issue in infection prevention, and the combination of real-world sampling with controlled spore experiments is a valuable approach.

That said, the manuscript requires significant revision to improve clarity, methodological rigor, and scientific presentation.

Major comments

Manuscript completeness and clarity

Comment: The current version appears incomplete and contains numerous fragmented sentences, missing values, and formatting issues (e.g., missing units, incomplete log reduction values, broken tables, and placeholder text). These issues make it difficult to fully assess the study. Carefully revise the entire manuscript for completeness. Ensure all numerical data (e.g., log reductions, concentrations, volumes) are clearly reported with correct units. Reconstruct tables and figures so they are readable and interpretable.

Response: Thank you for your suggestions. We have revised the whole manuscript as suggested. We have reconstructed the figures and tables. Please see Figure 2 and Tables 1 and 2, completed the missing values and units and described volume with units etc.

Methodological transparency

Key methodological details are insufficient or unclear:

Comment: Clarify the sampling protocol (number of replicates, timing, handling procedures).

Response: Thank you for your comment. We have clarified all the requested information’s in the revised manuscript as following. (See page#05, line 105-123)

The ultrasound probes were collected (from total thirty subjects) after use in female (aged >18 years) an in vitro fertilization clinic (Connect IVF, Sydney, Australia) from February 26 to April 02, 2025. All the subjects completed the study. These probes had been sheathed with a non-sterile latex free probe cover (Sonologic) prior to use. After use, the sheath was removed, and sterile cotton swabs (Bacto Laboratories, Mount Pritchard, NSW, Australia) were passed twice over a 2-5 cm2 area from the probes including from the top notched, bottom notched, left side of the handle, right side of the handle of one probe, grooves (bent areas) and lense areas of other probe (randomly selected) to examine the numbers and types of microbes present on the endoscopes prior to disinfection. The ultrasound probes were then loaded into the Lumicare ONE High Level Disinfection UVC system (Lumicare 606A743U7Y, Sydney, Australia) and the system run for a standard time of 90 seconds. After disinfection, the ultrasound probe was removed, and the opposite side of each probes’ sites was swabbed as described above. The swabs were then put into AMIES transport media (Bacto Laboratories, Australia) and transported to the microbiology laboratory of the School of Optometry and Vision Science University of New South Wales (UNSW) in cold chain and processed within 4 hours of collection.

Comment: Provide precise details of the UV-C system (dose, wavelength, distance, exposure uniformity).

Response: The Lumicare system is an automated HLD system (the device does everything on its own, dose determination). The previous study demonstrates that the Lumicare UVC-LED system (Lumicare 606A743U7Y, Sydney, Australia) disinfected the ultrasound probes after 90 sec cycle and effectively eliminated microorganisms associated with them.

During a standard HLD cycle, UVC LEDs generate UVC irradiation (265 nm – 275 nm) across the entire surface of the ultrasound probe positioned within the enclosed disinfection chamber at an approximate distance of 3 cm. (See page#5, line 116-118)

Comment: Explain how contamination rates and “100% decontamination” were defined.

Response: We added the following information’s in the revised manuscript (See page#11, line 203-206)

1. Rate of contamination of specific site was defined based on the following formula

• Number of site positive for microbial growth/ total number of sites screened for bacterial growth× 100.

2. While 100 % decontamination for specific site was defined based on the following formula

• Number of site negative for microbial growth/ total number of sites screened for bacterial growth× 100.

Comment: Describe statistical analysis more clearly (e.g., how P values were calculated with small sample sizes). Sample size and study design limitations.

Response: Due to the small sample size, the data were not assessed for normal distribution and were therefore analysed using non-parametric statistical tests to determine the P value. This study also has several general limitations, including sample collection from a single laboratory and the limited number of probes with complex geometries evaluated. These factors constrained both the sample size and the overall study design, which may limit the generalisability of the findings. These details have been added in the revised manuscript. Please see Page# 8, Line# 179-182.

Comment: The study is based on only two probes, which limits generalizability. This limitation should be explicitly acknowledged and discussed in more depth.

Response: We have included the following details in the revised manuscript. Please see Page#19-20, Line#407-417.

Another key limitation of this study was the inclusion of only two probes from a single probe category. Ultrasound probes are broadly classified into endocavity probes (e.g., transvaginal and transrectal) and external probes (e.g., linear, curved, and phased array) (1). The present study evaluated the effectiveness of the Lumicare UVC-LED system only against endocavity probes, using representative commonly used transvaginal/transrectal ultrasound probes manufactured by Mindray. However, each probe type contains unique and complex surface geometries that require independent evaluation for effective disinfection. Consequently, this study provides limited insight into the disinfection performance across the broader range of ultrasound probe types. Therefore, future studies should evaluate the effectiveness of the Lumicare UVC system on multiple probe types with varying structural complexities.

Comment: Avoid overgeneralizing conclusions to broader clinical settings.

Response: Thank you for your suggestion. We have rewritten the conclusion as following. Please see Page#20, Line#418-422.

In conclusion, the findings demonstrate that the Lumicare UVC-LED system can effectively disinfect both straight and curved transvaginal probes by inactivating microorganisms residing in complex probe sites such as notches, handle, grooves, and lense areas etc. Furthermore, UVC treatment achieved consistent and substantial (> 6 log₁₀) reduction of B. subtilis spores across all tested complex sites, highlighting its potential to inactivate highly resistant microorganisms.

Data consistency and interpretation

Comment: There are inconsistencies and unclear reporting in the results:

Log reduction values are incomplete or inconsistently presented (e.g., “>6.7 l”, missing units). Some numerical results appear truncated or misaligned.

Response: Thank you for your suggestions. We have added unites with all the values.

Comment: Ensure consistency between text, tables, and figures.

Response: We have now rewritten everything consistently throughout text, tables, and figures.

Overstatement of conclusions

Comment: While the results are promising, statements such as “100% decontamination” and “complete elimination” should be interpreted cautiously:

Response: Thank you for your suggestions. We have carefully edited the words 100% decontamination. We have also deleted the words “complete elimination” and changed to “effectively elimination” wherever applicable.

Thank you for your suggestion we have changed words “complete elimination” to “decontamination: wherever applicable throughout the revised manuscript.

Comment: Discuss detection limits and potential residual contamination. Avoid implying absolute sterility unless this is methodologically justified.

Response: We greatly acknowledge your suggestions. We have removed the wordings “detection limits and potential residual contamination” and rewritten the sentence as “The small spore’s counts (0.23–0.33 log₁₀ CFU) may be attributable to the detection of organisms near the assay’s detection limit.”

Literature context and novelty

The discussion would benefit from clearer positioning within existing literature:

Comment: Expand comparison with previous studies on UV-C disinfection of ultrasound probes.

Response: A previous study reported the detection of Human Papillomavirus (HPV) on vaginal ultrasound probes and demonstrated that the virus was completely inactivated by a UVC disinfection system within a 90 sec disinfection cycle (2). Page #19, Line #403-405

In a previous study, a UVC device achieved a 2.17 log₁₀ reduction of S. aureus, E. faecalis, and E. coli within 5–7 min when disinfecting probes (3). The longer disinfection time (5–7 min) reported in the previous study, compared with the 90-second cycle in the recent study, may be attributed to probe disinfection being performed in the presence of ultrasound gel. In contrast, the gel was completely removed prior to disinfection in the present study. The presence of gel may have impeded UVC penetration, thereby reducing disinfection efficiency and necessitating a longer exposure time. Page #17, Line #335-341

A previous study reported that 3D scanning of endocavity bi-plane and curved array probes demonstrated an uneven distribution of irradiance across probe regions when exposed to a UVC system (4), which may compromise the performance of the disinfectant system. However, the current UVC-LED device incorporating UVMESH technology achieved complete microbial inactivation, including on complex region of both probe types. This difference may be attributed to variations in study design, as the previous study primarily modelled or assumed irradiance distribution across differently designed probe surfaces rather than evaluating the real-world antimicrobial efficacy of the disinfection system. In contrast, the present study directly assessed microbial inactivation on probe surfaces under practical conditions. Additionally, probe design and geometry can also contribute to variations in irradiance distribution across specific probe sites (4). Page #16, Line #314-324.

Comment: Clarify what is novel about your system (e.g., UVMESH technology) beyond existing UV-C approaches.

Response: Thank you for asking clarification on UVMESH technology. We have added following paragraph in the revised manuscript. Please see Page#16, Line # 324-329.

This suggests that UVC-LEDs delivered UVC light effectively across the entire probe surface using UVMESH technology, resulting in the elimination of microorganisms from complex and uneven probe surfaces (5). This technology enhances spatial distribution and more uniform irradiance on entire area of target surfaces. Moreover, this approach specifically minimises the risk of small areas remaining untreated and maximises decontamination efficiency, even across complex surface architectures and geometries. Additionally, the system is engineered to provide controlled and reproducible UVC exposure conditions, potentially enhancing consistency in microbial inactivation outcomes.

Language and structure

The manuscript requires substantial language editing:

Comment: Improve grammar, sentence structure, and readability.

Response: The revised manuscript has been carefully edited for grammar and improved for readability.

Comment: Remove repetition and ensure logical flow between sections.

Response: All repetitions have been removed, and the sentences are now coherent.

Comment: Ensure consistent terminology (e.g., UVC vs. UV-C, probe regions).

Response: We have consistently used UVC by replacing UV-C and regions by sites in the body text of whole manuscript.

Minor comments

Comment: Clearly define all abbreviations at first use.

Response: The abbreviations when introduced first time in the manuscript are defined. Please see as below

1. Colony forming units (CFU)

2. Matrix-Assisted Laser Desorption/Ionization Time-of-Flight (MALDI-TOF)

3. α-cyano-4-hydroxycinnamic acid (HCCA)

4. Chocolate Blood agar (CBA)

5. Saboraud dextrose agar (SDA)

6. Phosphate Buffer Saline (PBS)

Comment: Standardize bacterial nomenclature (italicization, full names).

Response: All the bacterial names written with full name in the manuscript, have been italicized.

Comment: Ensure all references are properly formatted and complete.

Response: All the references have been reformatted according to the journal requirements.

Comment: Improve figure captions to be self-explanatory.

Response: We have rewritten the figure captions for better understanding. Please see Figure 2 caption.

Figure 2. Effect of UVC treatment on recovery of B. subtilis spores from different sites of probes. Spores’s viable counts (log₁₀ CFU/site) recovered from control (non UVC treated) and samples (UVC treated) are shown for the handle, bottom notch and lens (Probe A), and central bent side, left side of edge and lens of (Probe B). Recovery of spores from control sites were > 6.5 log₁₀ CFU/site, however after UVC treatment recovery of spores from samples sites decreased to < 0.5 log₁₀ CFU/site. This shows that UVC-LED system reduced > 6 log₁₀ bacillus spores on complex sites of both probes.

References

The following references were cited in support of our response.

1. Shokoohi H, Armstrong P, Tansek R. Emergency department ultrasound probe infection control: challenges and solutions. Open Access Emerg Med. 2015;7:1-9.

2. Pichon M, Lebail-Carval K, Billaud G, Lina B, Gaucherand P, Mekki Y. Decontamination of intravaginal probes infected by human papillomavirus (HPV) using UVC decontamination system. J of Clinic Med. 2019;8(11):1776.

3. Bloc S, Mercadal L, Garnier T, Komly B, Leclerc P, Morel B, et al. Evaluation of a new disinfection method for ultrasound probes used for regional anesthesia: ultraviolet C light. J Ultrasound Med. 2011;30(6):785-8.

4. Yeoh L, Cogar L, Barak M, Tan LY, Spargo G, Burdach J. UV-C disinfection of ultrasound probes: Challenges of uneven irradiance on complex surfaces. PLoS One. 2024;19(10):e0312931.

5. https://lumicare.one/uvmesh. (Accessed on 02/06/2026) [

Attachment Submitted filename: Reviewer 2.docx

# Peer review: Decision Letter 1

**DOI:** 10.1371/journal.pone.0346554.r003

**Authors:** Marcos Pileggi

13 Jul 2026

Dear Dr. Yasir,

Thank you for submitting your manuscript to PLOS ONE. After careful consideration, we feel that it has merit but does not fully meet PLOS ONE’s publication criteria as it currently stands. Therefore, we invite you to submit a revised version of the manuscript that addresses the points raised during the review process.

The authors should improve the organization of the manuscript in accordance with the reviewers' recommendations and provide a clearer articulation of the hypothesis under investigation.

Please submit your revised manuscript by Aug 27 2026 11:59PM. If you will need more time than this to complete your revisions, please reply to this message or contact the journal office at plosone@plos.org. When you're ready to submit your revision, log on to https://www.editorialmanager.com/pone/ and select the 'Submissions Needing Revision' folder to locate your manuscript file.

- A letter that responds to each point raised by the academic editor and reviewer(s). You should upload this letter as a separate file labeled 'Response to Reviewers'.
- A marked-up copy of your manuscript that highlights changes made to the original version. You should upload this as a separate file labeled 'Revised Manuscript with Track Changes'.
- An unmarked version of your revised paper without tracked changes. You should upload this as a separate file labeled 'Manuscript'.

If you would like to make changes to your financial disclosure, please include your updated statement in your cover letter. Guidelines for resubmitting your figure files are available below the reviewer comments at the end of this letter.

If applicable, we recommend that you deposit your laboratory protocols in protocols.io to enhance the reproducibility of your results. Protocols.io assigns your protocol its own identifier (DOI) so that it can be cited independently in the future. For instructions see: https://journals.plos.org/plosone/s/submission-guidelines#loc-laboratory-protocols. Additionally, PLOS ONE offers an option for publishing peer-reviewed Lab Protocol articles, which describe protocols hosted on protocols.io. Read more information on sharing protocols at https://plos.org/protocols?utm_medium=editorial-email&utm_source=authorletters&utm_campaign=protocols.

As the corresponding author, your ORCID iD is verified in the submission system and will appear in the published article. PLOS supports the use of ORCID, and we encourage all coauthors to register for an ORCID iD and use it as well. Please encourage your coauthors to verify their ORCID iD within the submission system before final acceptance, as unverified ORCID iDs will not appear in the published article. *Only*  the individual author can complete the verification step; PLOS staff *cannot*  verify ORCID iDs on behalf of authors.

We look forward to receiving your revised manuscript.

Kind regards,

Marcos Pileggi, Ph.D

Academic Editor

PLOS One

Journal Requirements:

1. If the reviewer comments include a recommendation to cite specific previously published works, please review and evaluate these publications to determine whether they are relevant and should be cited. There is no requirement to cite these works unless the editor has indicated otherwise.

2. Please review your reference list to ensure that it is complete and correct. If you have cited papers that have been retracted, please include the rationale for doing so in the manuscript text, or remove these references and replace them with relevant current references. Any changes to the reference list should be mentioned in the rebuttal letter that accompanies your revised manuscript. If you need to cite a retracted article, indicate the article’s retracted status in the References list and also include a citation and full reference for the retraction notice.

[Note: HTML markup is below. Please do not edit.]

Reviewers' comments:

Reviewer's Responses to Questions

**Comments to the Author**

Reviewer #3: All comments have been addressed

Reviewer #4: (No Response)

**********

2. Is the manuscript technically sound, and do the data support the conclusions?

Reviewer #3: Partly

Reviewer #4: Partly

**********

3. Has the statistical analysis been performed appropriately and rigorously?

Reviewer #3: I Don't Know

Reviewer #4: Yes

**********

4. Have the authors made all data underlying the findings in their manuscript fully available?

The PLOS Data policy

Reviewer #3: Yes

Reviewer #4: Yes

**********

5. Is the manuscript presented in an intelligible fashion and written in standard English?

Reviewer #3: Yes

Reviewer #4: Yes

**********

Reviewer #3: In Manuscript PONE-D-26-13842_R1, Yasir and Willcox present their research on disinfection of transvaginal ultrasound probes with a UVC-LED based system. Overall, the revision presents solid data on this topic, but the manuscript lacks a certain flow with respect to the presentation of their data. In its current form, the detailed data presented in the Results section often makes reading the manuscript difficult. Overall, the paper is straightforward, it is more confusing than it needs to be.

- Transvaginal probes are dirty and become contaminated with routine use

- Clinical and disinfection rooms are dirty and may contribute to contamination

- Current cleaning methods are ineffective (however, there is no proof of this in the current data, it is implied)

- A new UVC disinfection method can significantly reduce contamination as shown by in vitro and ex vivo testing

I have put suggestions below:

1. Each section of the Results (e.g., Ex vivo testing), the reason behind the study is not presented. For example, On Page 22 Line 191, the section starts with a statement that “areas of probes A and B were heavily contaminated with microbes…” A statement such as, “To demonstrate the level of contamination on transvaginal ultrasound probe, various areas of the probes were swabbed and examined for the presence of bacteria.”

Each section would benefit by walking the reader through the flow of the experiments.

2. The detailed analysis of contamination by various bacterial species (page 23 Line 195-206) is hard to read. Do the authors believe that the different notches, sides of the handle etc., are contaminated differently because how they are used? Or are these areas just difficult to clean portions of the probe? It is fine to list the details about log10CFU for different species in the table, but the paragraph is hard to read. I think that just mentioning the variety of species found and the range of contamination (log10CFU) in Table 1 would be sufficient.

Giving detailed species for different regions of the probes suggests that the design is important. I believe that the authors are attempting to demonstrate the high level of contamination before disinfection and the variety of species. The level of detail presented would suggest there is more to the story.

3. The authors describe the rate of contamination of various portions of the probes (Page 25 Line 209-222) and this section is much easier to read/follow. Similarly, a simpler presentation of the data mentioned above in Comment 2, would improve the paper.

4. Page 26 Line 238 This section could also use an introductory statement, such as “probe covers are often utilized to prevent contamination of probes, but they could also serve as a source of infections.” Similarly, remove the log10CFU in the written paragraph and refer readers to the table for those values. Again, contamination is contamination but do the different species matter or is again just demonstration of the level of contamination.

Probe covers were examined on the inside so again the level of contamination is impressive but would be expected.

5. For the probes/covers examined with ex vivo testing, it appears that they are heavily contaminated. How were they cleaned prior to use? Were they cleaned using standard protocols? Did the authors demonstrate that they were clean prior to use? In other words, does this contamination represent the “cleaned” probes that were assumed to be cleaned by current methods (non-UVC)? If so, then the authors need to comment on the quality of current sterilization methods, because this is the impetus for the need for a new sterilization method or UVC.

6. On Page 21 Line 168-9, the authors state that probes were disinfected with 4% ethanol. Is this the standard sterilization process? The authors need to give the reader more background about standard sterilization. If current methods are failing or had been assumed to be adequate, then again, the need for a new disinfection method is obvious. The authors are missing opportunities to make their current work relevant and more importantly necessary for disinfection.

7. Is there any published data about cross-infection between patients following the use of a transvaginal ultrasound probe? Yes, probes are dirty following their use, but what does it look like after standard cleaning? The paper would be enhanced if the authors had compared standard disinfection with the new UVC based method. I am not asking for more experiments to be done, but if this data is available, then the paper would be greatly strengthened in relevance and importance.

8. The authors should include a figure of the UVC-LED based system for completeness.

9. For the In vitro testing section (page 28 Line 271-291), again an opening introductory line describe the experiment would help the flow of the Results section. For example, “In order to determine the efficacy of UVC treatment, bleach sterilized probes were inoculated with control bacteria and the reduction in recovered bacteria were measured before and after UVC treatment.”

Again, does specific efficacy of UVC treatment on various sites (e.g., lens, central bent side etc.) matter? The authors are not attempting to comment on the design of the probe, just that certain regions with 3D structures are more prone to contamination. I am not sure such details matter for the study and could be shortened, removed, or the range of log10 reductions could be presented.

10. The analysis of baseline contamination of the clinical and disinfectant rooms is relevant to the study, but why was air sampling not used instead? Open agar plates were left in the room, but a more complete analysis of room contamination is often done with an SAS 180 Impaction Air Sampler. Again, not asking for more experiments at this stage of submission, but for future studies, I would suggest use of such a device.

I would move this section to either the end of Results or I would consider adding it as a supplemental table.

11. In the Discussion (page 32 Line 374-390), the authors discuss at length the various types of bacteria found on probes and in the rooms. The detailed discussion about different types of bacteria common to both sites is of interest, but not as much as their results on cleaning the probes/disinfection system. Since there was no proof of cross-contamination between patients, this section is purely hypothetical and doesn’t add to their major findings.

Reviewer #4: PLOS One

Inactivation of Microorganisms in the Complex Regions of Transvaginal Ultrasound

Probes By a UVC-LED Light Based Disinfection System

The comments of previous reviewers have been answered in the revised version.

Major points

L111 How was the sampling standardised for example same operator, same pressure applied, same area? It is stated the area sampled was between 2 and 5 cm2. How were other areas selected randomly, was there a computer-generated sequence or simply the choice of operator? Was sampling reproducibility assessed? Alginate swabs would have a higher yield. Was any soil visible on sheaths?

L117 Was the same machine used in all cases?

L127 The area swabbed was between 2 and 5 cm2 so how was reproducibility ensured?

L134 Was the recovery of organisms checked for reproducibility or were replicates used?

L143 Settle plates are not quantitative so do not indicate the likelihood of contamination. Were staff wearing PPE and were rooms ventilated as that would affect results.

L171 Although B subtilis is a standard test of disinfection, it is not an organism associated with clinical use. Why were bacterial or viral pathogens not used that would be expected to contaminate ultrasound probes? The likelihood of killing of pathogens for example HPV or C difficile spores (from faecal contamination) should be considered (HPV is mentioned in the discussion).

L195 The level of contamination already on probes despite the use of a sheath was striking. These had not been decontaminated before swabbing but was there any delay before swabbing? What decontamination was used routinely? The variety of species suggests contamination from skin, water and environment rather than vaginal or faecal flora. Hence this maybe mainly operator rather than patient contamination. The findings are difficult to generalise because only two probes were used and they were of different designs.

L242 The sheaths show skin flora rather than vaginal flora which may reflect insertion only of the tip of the sheath into the vaginal or delay between use and sampling.

L266 Environmental airborne flora are less relevant than those acquired by direct contact with the probes. Similar common species on settle plates and probes e.g. S epidermidis do not infer probe isolates came from air. Direct contact is much more likely.

L278 UVC was effective but does repeated use have a damaging effect on plastics? Turnaround times in clinic would be of interest.

L395 the discussion could be shortened.

Minor points

L74 gonorrhoeae not gonorrhoea

L112 lens not lense

L133/5 Becton Dickinson not Beckton Dekickinson

L319 different

L404 spore counts

**********

what does this mean?). If published, this will include your full peer review and any attached files.

If you choose “no”, your identity will remain anonymous but your review may still be made public.

**Do you want your identity to be public for this peer review?**  For information about this choice, including consent withdrawal, please see our Privacy Policy

Reviewer #3: **Yes:**  Timothy Angelotti MD PhD

Reviewer #4: No

**********

[NOTE: If reviewer comments were submitted as an attachment file, they will be attached to this email and accessible via the submission site. Please log into your account, locate the manuscript record, and check for the action link "View Attachments". If this link does not appear, there are no attachment files.]

To ensure your figures meet our technical requirements, please review our figure guidelines: https://journals.plos.org/plosone/s/figures

You may also use PLOS’s free figure tool, NAAS, to help you prepare publication quality figures: https://journals.plos.org/plosone/s/figures#loc-tools-for-figure-preparation.

NAAS will assess whether your figures meet our technical requirements by comparing each figure against our figure specifications.

# Author comment: Author response to Decision Letter 2

**DOI:** 10.1371/journal.pone.0346554.r004

26 Aug 2026

Reviewer #3:

In Manuscript PONE-D-26-13842_R1, Yasir and Willcox present their research on disinfection of transvaginal ultrasound probes with a UVC-LED based system. Overall, the revision presents solid data on this topic, but the manuscript lacks a certain flow with respect to the presentation of their data. In its current form, the detailed data presented in the Results section often makes reading the manuscript difficult. Overall, the paper is straightforward, it is more confusing than it needs to be.

- Transvaginal probes are dirty and become contaminated with routine use

- Clinical and disinfection rooms are dirty and may contribute to contamination

- Current cleaning methods are ineffective (however, there is no proof of this in the current data, it is implied)

- A new UVC disinfection method can significantly reduce contamination as shown by P in vitro and ex vivo testing

I have put suggestions below:

Comment: Each section of the Results (e.g., Ex vivo testing), the reason behind the study is not presented. For example, On Page 22 Line 191, the section starts with a statement that “areas of probes A and B were heavily contaminated with microbes…” A statement such as, “To demonstrate the level of contamination on transvaginal ultrasound probe, various areas of the probes were swabbed and examined for the presence of bacteria.”

Each section would benefit by walking the reader through the flow of the experiments.

Response: Thank you for your suggestions. We have added similar in each section wherever required. Such as;

1. To demonstrate the level of contamination on transvaginal ultrasound probe, various areas of the probes were swabbed and examined for the presence of bacteria.

2. To confirm that each probe was effectively disinfected prior to spores’ inoculation before each experimental repeat, 4% bleach was used.

3. In order to determine the efficacy of UVC treatment, bleach (4%) sterilized probes were inoculated with control bacillus spores and the reduction in recovered spores were measured before and after UVC treatment.

Comment: The detailed analysis of contamination by various bacterial species (page 23 Line 195-206) is hard to read. Do the authors believe that the different notches, sides of the handle etc., are contaminated differently because how they are used? Or are these areas just difficult to clean portions of the probe? It is fine to list the details about log10CFU for different species in the table, but the paragraph is hard to read. I think that just mentioning the variety of species found and the range of contamination (log10CFU) in Table 1 would be sufficient.

Giving detailed species for different regions of the probes suggests that the design is important. I believe that the authors are attempting to demonstrate the high level of contamination before disinfection and the variety of species. The level of detail presented would suggest there is more to the story.

Response: Thank you for giving your valuable suggestions.

• We have adjusted the paragraph as suggested. We have removed the information’s from paragraph and retained in the Table 1.

• Different regions of the probes such as notches, groves, sides of the handle are difficult to clean areas of the probes. Therefore, we have chosen to determine the disinfectant performance of UVC-LED system against the microorganisms recovered from these complex and difficult to clean areas. (please see page# 15, Line#329-330)

• We have updated this information about (the range of contamination (log10CFU)) in the revised manuscript. (please see page# 9, Line#215 and 220)

Comment: The authors describe the rate of contamination of various portions of the probes (Page 25 Line 209-222) and this section is much easier to read/follow. Similarly, a simpler presentation of the data mentioned above in Comment 2, would improve the paper.

Response: Thank you for your suggestions. As suggested, we have rewritten and shortened the paragraph.

Comment: Page 26 Line 238 This section could also use an introductory statement, such as “probe covers are often utilized to prevent contamination of probes, but they could also serve as a source of infections.” Similarly, remove the log10 CFU in the written paragraph and refer readers to the table for those values. Again, contamination is contamination but do the different species matter or is again just demonstration of the level of contamination.

Probe covers were examined on the inside so again the level of contamination is impressive but would be expected.

Response: As suggested, we have added

1. “probe covers are often utilized to prevent contamination of probes, but they could also serve as a source of infections.” We have removed log10 CFU values written in the paragraph and wrote “refer to Table 3” in the paragraph. (please see page# 12, Line#253 and 254)

2. Yes, agree with your opinion that contamination is contamination either one or many bacteria and with single CFU or many CFUs. However, we have described bacterial contamination, including the identification of species and their respective levels to maintain consistency throughout the manuscript and to provide a comprehensive representation of the microbial profiles observed across all data obtained in this study.

3. The contamination of probe cover is expected. In previous study Yasir and Willcox (1), we have determined that probe covers from inside was contaminated with Bacillus subtilis.

We have included following line in the revised manuscript to support our statement.

4. Previous study demonstrated that probe covers were contaminated with Bacillus subtilis with level up to 2.8 log10 CFU (1). (please see page# 18, Line#398 and 399)

Comment: For the probes/covers examined with ex vivo testing, it appears that they are heavily contaminated. How were they cleaned prior to use? Were they cleaned using standard protocols? Did the authors demonstrate that they were clean prior to use? In other words, does this contamination represent the “cleaned” probes that were assumed to be cleaned by current methods (non-UVC)? If so, then the authors need to comment on the quality of current sterilization methods, because this is the impetus for the need for a new sterilization method or UVC.

Response: Thank you for seeking clarification. Please find our response below.

• Prior to in vivo use in the clinic, probes were sheathed with non-sterile, latex-free probe covers, and coated with non-sterile gel. Also no wipe-down or manual cleaning step was performed between removal of the sheath and swab sampling — this was a deliberate study design choice to capture worst-case contamination. (please see page# 6, Line#138 and 139)

• Yes, this is the key methodological point in this study we deliberately followed the standard procedures undertaken within an Ultrasound department prior to HLD. This allowed us to test the UVC system under standard real-world conditions. In the standard protocol the probes were wiped over with a paper towel to remove debris.

• In routine clinical practice, ultrasound probes are cleaned with paper towel or wipes to remove visible contamination, including debris and organic material. However, these cleaning methods may not achieve complete removal or inactivation of microorganisms on the probe surface. Therefore, for semi-critical devices, HLD is required following the cleaning step as part of standard infection prevention and control protocols.

Comment: On Page 21 Line 168-9, the authors state that probes were disinfected with 4% ethanol. Is this the standard sterilization process? The authors need to give the reader more background about standard sterilization. If current methods are failing or had been assumed to be adequate, then again, the need for a new disinfection method is obvious. The authors are missing opportunities to make their current work relevant and more importantly necessary for disinfection.

Response: Our response is below.

• We apologise for this error. In the in vitro experiments, the probes were treated with 4% sodium hypochlorite (bleach) to achieve repeated sterility between experimental runs. This procedure was used specifically to ensure that the probes were free of viable microorganisms before subsequent inoculation with spores. We acknowledge that treatment with 4% sodium hypochlorite is not a standard sterilisation method for clinical ultrasound probes and was used solely as part of the experimental protocol for the in vitro study. However, this is not recommended by probe manufacturers due to the potential for damage to the metal and plastic components of the probes (2). (See page# 18, Line#410-412).

• Various methods, including chemical soaking, chemical aerosolisation, and surface wiping, have been employed for ultrasound probe disinfection. Chemical soaking requires the probe to be immersed in a disinfectant solution for approximately 8–45 minutes, whereas chemical aerosolisation involves nebulisation of hydrogen peroxide within a device chamber and may require approximately 7–12 minutes, depending on the system and operating conditions. Quaternary ammonium compounds and chlorhexidine in solution (3), or soaked with wipes (4) found ineffective against HPV. Similarly, glutaraldehyde exhibits broad-spectrum antimicrobial activity, but its use is limited due to potential damage to ultrasound probes (5). These chemical-based approaches may present occupational and environmental concerns due to potential exposure to chemical disinfectants. (See page# 4, Line#83- 93).

Comment: Is there any published data about cross-infection between patients following the use of a transvaginal ultrasound probe? Yes, probes are dirty following their use, but what does it look like after standard cleaning? The paper would be enhanced if the authors had compared standard disinfection with the new UVC based method. I am not asking for more experiments to be done, but if this data is available, then the paper would be greatly strengthened in relevance and importance.

Response:

1. The real risk of infection associated with TVUS probes following use without adequate decontamination is unknown. No case of related specific infection (cross-contamination between patients) has been reported in the literature, but contaminated ultrasound transducers can be potential vector for transfer of microorganisms.

2. The cleaning process, which precedes disinfection, is crucial in removing organic matter that can interfere with the effectiveness of disinfectants. Inadequate cleaning can result in residual contaminants that provide a protective barrier for pathogens, rendering disinfection ineffective. This scenario can lead to the transmission of infections, posing serious risks to patients.

3. According to the Australasian Society for Ultrasound in Medicine (ASUM) and Australian Standard (AS5369), American Institute of Ultrasound in Medicine (AIUM) guidelines, transvaginal ultrasound probes are classified as semi-critical devices and require high-level disinfection (HLD) following cleaning. Various disinfectants have been used for probe disinfection, including quaternary ammonium compounds, chlorhexidine, and quaternary ammonium-based wipes. Although these agents are effective against a range of bacteria and fungi within 5-20 minutes, their effectiveness against certain viruses, including HPV, may be limited (3, 4). Sherman et al. reported that 70% alcohol was highly effective against bacterial contamination but demonstrated limited activity against viral contamination. Similarly, glutaraldehyde, ortho-phthalaldehyde, and 4% chlorhexidine gluconate were found to be ineffective against HPV16 (6, 7). However, UV-C devices can disinfect vaginal ultrasound probes faster and can inactivate all the microorganism including viruses (8, 9). (See page# 16, Line#344-355)

In addition, chemical disinfectants may also damage to surface of ultrasound probe. Although sodium hypochlorite has broad-spectrum antimicrobial activity, its potential for surface damage limits its use for probe disinfection (2). In contrast, independent testing demonstrated that repeated exposure to the Lumicare UV-C LED system did not adversely affect probe surface. (See page# 18, Line#410-412) and (See page#19 , Line#428-431).

Comment: The authors should include a figure of the UVC-LED based system for completeness.

Response: Thank you for your suggestions. We have added diagram UVC Lumicare device. (See Figure 1)

Figure: 2. Lumicare UVC-LED based system: frontal view (A) and chamber view showing the probes suspended inside the chamber (B).

Comment: For the In vitro testing section (page 28 Line 271-291), again an opening introductory line describe the experiment would help the flow of the Results section. For example, “In order to determine the efficacy of UVC treatment, bleach sterilized probes were inoculated with control bacteria and the reduction in recovered bacteria were measured before and after UVC treatment.”

Again, does specific efficacy of UVC treatment on various sites (e.g., lens, central bent side etc.) matter? The authors are not attempting to comment on the design of the probe, just that certain regions with 3D structures are more prone to contamination. I am not sure such details matter for the study and could be shortened, removed, or the range of log10 reductions could be presented.

Response: Thank you for your suggestions we have added the following statement.

1. “In order to determine the efficacy of UVC treatment, bleach sterilized probes were inoculated with control bacteria and the reduction in recovered bacteria were measured before and after UVC treatment.” (Please see Page #13, Line# 280-282)

2. We have discussed and commented on the design of the probe in discussion section (Please see Page #20, Line# 452-461)

3. All authors agreed to retain the detailed Log₁₀ values for each site of the probe in the Results section of the revised manuscript.

Comment: The analysis of baseline contamination of the clinical and disinfectant rooms is relevant to the study, but why was air sampling not used instead? Open agar plates were left in the room, but a more complete analysis of room contamination is often done with an SAS 180 Impaction Air Sampler. Again, not asking for more experiments at this stage of submission, but for future studies, I would suggest use of such a device.

I would move this section to either the end of Results or I would consider adding it as a supplemental table.

Response:

1. As suggested, we have removed this table from main part of manuscript and added as Supplementary Table 1.

2. The air sampler will be used in future studies to investigate the relationship between environmental microbial contamination and ultrasound probe contamination.

We have also included the following statement in the revised manuscript.

3. While environmental contamination was documented but its transmission dynamics was not determined, future research should investigate the transmission dynamics with an SAS 180 Impaction Air Sampler. (Please see Page #19-20, Line#440-442)

Comment: In the Discussion (page 32 Line 374-390), the authors discuss at length the various types of bacteria found on probes and in the rooms. The detailed discussion about different types of bacteria common to both sites is of interest, but not as much as their results on cleaning the probes/disinfection system. Since there was no proof of cross-contamination between patients, this section is purely hypothetical and doesn’t add to their major findings.

Response: Thank you for your suggestions. We have reduced the details in this paragraph (Please see Page #19, Line#436-440).

References

1. Yasir M, Willcox MDP. The use of ultraviolet light generated from light-emitting diodes for the disinfection of transvaginal ultrasound probes. PLoS One. 2024;19(2):e0298449.

2. Abramowicz JS, Evans DH, Fowlkes JB, Maršal K, terHaar G. Guidelines for Cleaning Transvaginal Ultrasound Transducers Between Patients. Ultra in Med & Biol. 2017;43(5):1076-9.

3. M'Zali F, Bounizra C, Leroy S, Mekki Y, Quentin-Noury C, Kann M. Persistence of microbial contamination on transvaginal ultrasound probes despite low-level disinfection procedure. PloS one. 2014;9(4):e93368.

4. Casalegno J-s, Le Bail Carval K, Eibach D, Valdeyron M-L, Lamblin G, Jacquemoud H, et al. High risk HPV contamination of endocavity vaginal ultrasound probes: an underestimated route of nosocomial infection? PloS one. 2012;7(10):e48137.

5. Zambrano-Carrillo MF, Hernandez-Jerez AF. Occupational risk assessment of glutaraldehyde through personal air monitoring in a hospital setting. Ann of Work Expos and Health. 2024;68(1):97-103.

6. Meyers J, Ryndock E, Conway MJ, Meyers C, Robison R. Susceptibility of high-risk human papillomavirus type 16 to clinical disinfectants. J Antimicrob Chemother. 2014;69(6):1546-50.

7. Ienghong K, Kleebbuakwan K, Apiratwarakul K, Phungoen P, Gaysonsiri D, Bhudhisawasdi V. Comparison of Cleaning Methods for Ultrasound Probes at an Emergency Department in a Resource-Limited Country. J of the Med Asso of Thai. 2020;103:67.

8. Meyers C, Milici J, Robison R. UVC radiation as an effective disinfectant method to inactivate human papillomaviruses. PLoS One. 2017;12(10):e0187377.

9. Pichon M, Lebail-Carval K, Billaud G, Lina B, Gaucherand P, Mekki Y. Decontamination of intravaginal probes infected by human papillomavirus (HPV) using UVC decontamination system. J of Clinic Med. 2019;8(11):1776.

Reviewer #4: PLOS One

Inactivation of Microorganisms in the Complex Regions of Transvaginal Ultrasound

Probes By a UVC-LED Light Based Disinfection System

The comments of previous reviewers have been answered in the revised version.

Major points

Comments: L111 How was the sampling standardised for example same operator, same pressure applied, same area? It is stated the area sampled was between 2 and 5 cm2. How were other areas selected randomly, was there a computer-generated sequence or simply the choice of operator? Was sampling reproducibility assessed? Alginate swabs would have a higher yield. Was any soil visible on sheaths?

Response:

The sampling was standardised in the following ways :

• Single Operator/Collector - trained by the UNSW researcher for technique.

• Same pressure applied at the collection points - although this could not be quantified. Same technique.

• Sterile cotton swabs were passed twice over a 2–5 cm² area prior to disinfection, and the opposite side was swabbed after disinfection using the same technique. The area was initially marked to facilitate identification and was subsequently applied consistently to all probes throughout the study.

We have added the following information’s in the revised manicurist.

All the sampling was performed by a single IVF clinic staff trained by the University of New South researchers in accordance with the standard operating procedures. The probe’s area on was initially marked to facilitate identification and was subsequently applied consistently to all probes throughout the study. (Page# 6, Line#132-136).

• We have done preliminary study for isolation and enumeration of microorganism using the cotton swabs in the AMIES transport media. We have obtained comparable results to those with alginate swabs in phosphate buffer saline. Depending upon the availability we used cotton swab in AMIES media.

• Some visible debris was present on the sheaths. Standard Ultrasound gel is applied to the tip of the transducer prior to covering it with the sheath (to ensure the transducer membrane is coupled appropriated to the sheath and in turn the cavity for appropriate Ultrasound transmission). (Page# 6, Line#125-126).

Comments: L117 Was the same machine used in all cases?

Response: To get the consistent effectiveness against all the probes, same machine was used to disinfect probes (Page# 8, Line#193-195). Photograph of machine with serial number is provided in the revised manuscript.

Comments: L127 The area swabbed was between 2 and 5 cm2 so how was reproducibility ensured?

Response: The area was initially marked to facilitate identification and was subsequently applied consistently to all probes throughout the study.

Comments: L134 Was the recovery of organisms checked for reproducibility or were replicates used?

Response: Due to nature of experiment or study, we were not able to sampling replicate using the same probe.

Comments: L143 Settle plates are not quantitative so do not indicate the likelihood of contamination. Were staff wearing PPE and were rooms ventilated as that would affect results.

Response: The study and sample collections were undertaken to reflect an environment that is a standard in use Ultrasound environment. PPE were worn by staff, apart from the standard air-conditioning. no ventilation was there in the room.

Comments: L171 Although B subtilis is a standard test of disinfection, it is not an organism associated with clinical use. Why were bacterial or viral pathogens not used that would be expected to contaminate ultrasound probes? The likelihood of killing of pathogens for example HPV or C difficile spores (from faecal contamination) should be considered (HPV is mentioned in the discussion).

Response: Our response is below.

• B. subtilis spores are considered highly resistant to disinfection. Several regulatory standards and methods, including EN, ASTM, and AOAC, recommend the use of B. subtilis spores for evaluating the efficacy of disinfection processes. Therefore, we selected B. subtilis spores as the challenge organism to evaluate the efficacy of the UVC device on probes.

• However, C. difficile spores will be evaluated in future studies to further assess the efficacy of the device against clinically relevant bacterial spores.

Accordingly, we have included the following statement in the revised manuscript.

“Future research should explore the effectiveness of Lumicare UVC-LED system against viral pathogens especially against HPV and spores of the Clostridium difficile (a faecal contaminates) (Page# 20, Line#450-452).

Comments: L195 The level of contamination already on probes despite the use of a sheath was striking. These had not been decontaminated before swabbing but was there any delay before swabbing? What decontamination was used routinely? The variety of species suggests contamination from skin, water and environment rather than vaginal or faecal flora. Hence this maybe mainly operator rather than patient contamination. The findings are difficult to generalise because only two probes were used and they were of different designs.

Response: The reviewer raises an important point regarding the timing of sampling and the impact of routine probe reprocessing.

• The ultrasound probes assessed in this study were sampled immediately following clinical use. Therefore, the microbial burden identified represents the contamination present at the commencement of the reprocessing workflow.

• The ultrasound probes are routinely cleaned with water and wipe over with a paper towel prior to HLD. (Page# 6, Line#135-137)

• We agree with the reviewer that potential environmental contamination cannot be completely excluded. However, previous studies (1-3) have reported the presence of similar microorganisms on transvaginal ultrasound probes, which may pose a potential risk of infection if the probes are not adequately cleaned and disinfected. (These references are updated in the revised manuscript)

Regarding “for limitation of using two probes” the following paragraph is written in the discussion of the revised manuscript.

• Another key limitation of this study was the inclusion of only two probes from a single probe category. Ultrasound probes are broadly classified into endocavity probes (e.g., transvaginal and transrectal) and external probes (e.g., linear, curved, and phased array) (4). The present study evaluated the effectiveness of the Lumicare UVC-LED system only against endocavity probes, using representative commonly used transvaginal/transrectal ultrasound probes manufactured by Mindray (China). However, each probe type contains unique and complex surface geometries that require independent evaluation for effective disinfection. Consequently, this study provides limited insight into the disinfection performance across the broader range of ultrasound probe types. Therefore, future studies should evaluate the effectiveness of the Lumicare UVC system on multiple probe types with varying structural complexities. (Page# 20, Line#451-461)

Comments: L242 The sheaths show skin flora rather than vaginal flora which may reflect insertion only of the tip of the sheath into the vaginal or delay between use and sampling.

Response: The sampling was performed by trained staff (trained by University of New South Sydney, Australia) working in the IVF clinic in accordance with the standard operating procedures. In addition, samples were collected immediately after probe use to minimise any changes in the microbial load.

Comments: L266 Environmental airborne flora are less relevant than those acquired by direct contact with the probes. Similar common species on settle plates and probes e.g. S epidermidis do not infer probe isolates came from air. Direct contact is much more likely.

Response: According to your suggestion and the comments from the other reviewer, we agree that the environmental flora are not directly relevant to the microorganisms associated with probe contamination. Therefore, we have moved these results to the Supplementary Table.

Comments: L278 UVC was effective but does repeated use have a damaging effect on plastics? Turnaround times in clinic would be of interest.

Response: Thanks for asking clarification on the this. The following response is added in the revised manuscript.

• As part of the regulatory evaluation of the Lumicare UVC-LED system, material compatibility testing confirmed that repeated exposure to UVC does not adversely affect probe integrity or performance. Lumicare device has completed material compatibility assessments through its regulatory pathways (CE Mark and ARTG registration), and compatibility has been confirmed with major ultrasound probe manufacturers, including the manufacturers of the probes evaluated in this study. These assessments demonstrated that repeated UVC-based high-level disinfection does not result in detrimental effects on probe materials. (Page# 19, Line#428-435)

Regarding turnaround time following information is added in the revised manuscript.

• In the clinical workflow. turnaround time between patients is an important consideration, particularly in high-throughput environments. Routine IVF ultrasound examinations are typically brief (5–10 minutes), requiring rapid reprocessing of probes between patients. The Lumicare UVC-LED system is designed to provide high-level disinfection within a timeframe compatible with clinical workflows, enabling probe availability for subsequent patients without significant disruption to clinic throughput. (Page# 4-5, Line#96-101)

References

1. Buescher DL, Möllers M, Falkenberg MK, Amler S, Kipp F, Burdach J, et al. Disinfection of transvaginal ultrasound probes in a clinical setting: comparative performance of automated and manual reprocessing methods. Ultra Obstet Gynecol. 2016;47(5):646-51.

2. Ejtehadi F, Ejtehadi F, Teb JC, Arasteh MM. A safe and practical decontamination method to reduce the risk of bacterial colonization of ultrasound transducers. J Clin Ultrasound. 2014;42(7):395-8.

3. M'Zali F, Bounizra C, Leroy S, Mekki Y, Quentin-Noury C, Kann M. Persistence of microbial contamination on transvaginal ultrasound probes despite low-level disinfection procedure. PLoS One. 2014;9(4):e93368.

4. Shokoohi H, Armstrong P, Tansek R. Emergency department ultrasound probe infection control: challenges and solutions. Emerg Med. 2015;7:1-9.

Attachment Submitted filename: Reviewer 3.docx

# Peer review: Decision Letter 2

**DOI:** 10.1371/journal.pone.0346554.r005

**Authors:** Marcos Pileggi

29 Sep 2026

Inactivation of Microorganisms in the Complex Regions of Transvaginal Ultrasound Probes By a UVC-LED Light Based Disinfection System

PONE-D-26-13842R2

Dear Dr. Yasir,

We’re pleased to inform you that your manuscript has been judged scientifically suitable for publication and will be formally accepted for publication once it meets all outstanding technical requirements.

Within one week, you’ll receive an e-mail detailing the required amendments. When these have been addressed, you’ll receive a formal acceptance letter and your manuscript will be scheduled for publication.

An invoice will be generated when your article is formally accepted. Please note, if your institution has a publishing partnership with PLOS and your article meets the relevant criteria, all or part of your publication costs will be covered. Please make sure your user information is up-to-date by logging into Editorial Manager at Editorial Manager® and clicking the ‘Update My Information' link at the top of the page. For questions related to billing, please contact billing support.

If your institution or institutions have a press office, please notify them about your upcoming paper to help maximize its impact. If they’ll be preparing press materials, please inform our press team as soon as possible -- no later than 48 hours after receiving the formal acceptance. Your manuscript will remain under strict press embargo until 2 pm Eastern Time on the date of publication. For more information, please contact onepress@plos.org.

Kind regards,

Marcos Pileggi, Ph.D

Academic Editor

PLOS One

Additional Editor Comments (optional):

Reviewers' comments:

Reviewer's Responses to Questions

**Comments to the Author**

Reviewer #3: All comments have been addressed

Reviewer #4: All comments have been addressed

**********

2. Is the manuscript technically sound, and do the data support the conclusions?

Reviewer #3: Yes

Reviewer #4: Yes

**********

3. Has the statistical analysis been performed appropriately and rigorously?

Reviewer #3: Yes

Reviewer #4: Yes

**********

4. Have the authors made all data underlying the findings in their manuscript fully available?

The PLOS Data policy

Reviewer #3: Yes

Reviewer #4: Yes

**********

5. Is the manuscript presented in an intelligible fashion and written in standard English?

Reviewer #3: Yes

Reviewer #4: Yes

**********

Reviewer #3: The manuscript has been edited appropriately according to comment from the reviewers. I have no further comments.

Reviewer #4: The comments have been addressed appropriately.

However, L134, if reproducibility of sampling could not be determined it is important to state it could not be measured due to the nature of the sampling.

L280 Suggest sodium hypochlorite not ‘bleach’

**********

what does this mean?). If published, this will include your full peer review and any attached files.

If you choose “no”, your identity will remain anonymous but your review may still be made public.

**Do you want your identity to be public for this peer review?**  For information about this choice, including consent withdrawal, please see our Privacy Policy

Reviewer #3: **Yes:**  Timothy Angelotti MD PhD

Reviewer #4: **Yes:**  Prof APR Wilson

**********

# Editor report: Acceptance letter

**DOI:** 10.1371/journal.pone.0346554.r006

**Authors:** Marcos Pileggi

PONE-D-26-13842R2

PLOS One

Dear Dr. Yasir,

I'm pleased to inform you that your manuscript has been deemed suitable for publication in PLOS One. Congratulations! Your manuscript is now being handed over to our production team.

At this stage, our production department will prepare your paper for publication. This includes ensuring the following:

* All references, tables, and figures are properly cited

* All relevant supporting information is included in the manuscript submission,

* There are no issues that prevent the paper from being properly typeset

You will receive further instructions from the production team, including instructions on how to review your proof when it is ready. Please keep in mind that we are working through a large volume of accepted articles, so please give us a few days to review your paper and let you know the next and final steps.

Lastly, if your institution or institutions have a press office, please let them know about your upcoming paper now to help maximize its impact. If they'll be preparing press materials, please inform our press team within the next 48 hours. Your manuscript will remain under strict press embargo until 2 pm Eastern Time on the date of publication. For more information, please contact onepress@plos.org.

You will receive an invoice from PLOS for your publication fee after your manuscript has reached the completed accept phase. If you receive an email requesting payment before acceptance or for any other service, this may be a phishing scheme. Learn how to identify phishing emails and protect your accounts at https://explore.plos.org/phishing.

If we can help with anything else, please email us at customercare@plos.org.

Thank you for submitting your work to PLOS One and supporting open access.

Kind regards,

PLOS One Editorial Office Staff

on behalf of

Dr. Marcos Pileggi

Academic Editor

PLOS One
